# Supplementary material for: Comparative studies of 2168 plasma proteins measured by two affinity-based platforms in 4000 Chinese adults
Source: Nat Commun. 2025 Feb 21;16:1869. doi: 10.1038/s41467-025-56935-2 (PMC11845630; doi:10.1038/s41467-025-56935-2)
Supplement: Supplementary file 1 — Supplementary Information [file 41467_2025_56935_MOESM1_ESM.pdf]

Comparative studies of genetic and phenotypic associations for 2,168 plasma proteins measured by two affinity-based platforms in 4,000 Chinese adults

Baihan Wang, Alfred Pozarickij, Mohsen Mazidi, Neil Wright, et al.

Contents

Supplementary Note 1: Members of CKB Collaborative Group 4
Supplementary Note 2: Reagent matching based on observational correlations 5
Supplementary Note 3: Effect of ambient temperature and time since last meal on protein levels 6
Supplementary Figure 1: Correlations of levels of 2,168 proteins (2,749 pairs) between Olink and SomaScan 7
Supplementary Figure 2: Pearson's r of levels of 2,168 proteins (2,749 pairs) between Olink and SomaScan 8
Supplementary Figure 3: Correlations of levels of 1,694 one-to-one matched proteins between Olink and SomaScan 9
Supplementary Figure 4: Pearson's r of levels of 1,694 proteins between Olink and SomaScan 10
Supplementary Figure 5: Correlations of levels of 1,694 proteins between Olink and SomaScan in 2,025 subcohort participants 11
Supplementary Figure 6: Spearman's rho of all possible regent pairs between Olink and SomaScan for 2,168 proteins 12
Supplementary Figure 7: Features predictive of correlations in Boruta feature selection 13
Supplementary Figure 8: Correlations between protein levels measured by Olink and SomaScan platforms according to degree of dilution in Olink assay 14
Supplementary Figure 9: Correlations between protein levels measured by Olink and SomaScan platforms according to degree of dilution in SomaScan assay 15
Supplementary Figure 10: number of pQTLs identified in each platform 16
Supplementary Figure 11: Number of proteins with *cis*-pQTLs discovered in Olink and SomaScan and colocalisation results 17
Supplementary Figure 12: Observational correlations between Olink and SomaScan and proteins with colocalising *cis*-pQTLs 18
Supplementary Figure 13: Factors explaining discordant findings on *cis*-pQTLs between Olink and SomaScan 19
Supplementary Figure 14: *cis*-pQTLs for ALDH2 identified in Olink and SomaScan platforms and colocalisation results 20
Supplementary Figure 15: *cis*-pQTLs for PLA2G7 identified in Olink and SomaScan platforms and colocalisation results 21

Supplementary Figure 16: *cis*-pQTLs for PCSK9 identified in Olink and SomaScan platforms and colocalisation results.......... 22

Supplementary Figure 17: Number of proteins significantly associated with BMI and their effect sizes after applying FDR correction for multiple testing.......... 23

Supplementary Figure 18: Number of proteins significantly associated with BMI and their effect sizes after applying Bonferroni correction for multiple testing.......... 24

Supplementary Figure 19: Comparison of effect sizes for proteins associated with BMI between Olink and SomaScan.......... 25

Supplementary Figure 20: Observational correlations and shared associations for BMI.......... 26

Supplementary Figure 21: Factors explaining discordant findings on BMI associations between Olink and SomaScan 27

Supplementary Figure 22: Variance explained by top 10 principal components in each platform 28

Supplementary Figure 23: Number of proteins significantly associated with selected baseline characteristics for Olink and SomaScan platforms.......... 29

Supplementary Figure 24: Concordance of associations of proteins with participant characteristics between Olink and SomaScan platforms.......... 30

Supplementary Figure 25: Correlation of effect sizes for proteins significantly associated with participant characteristics between Olink and SomaScan platforms 31

Supplementary Figure 26: Number of proteins significantly associated with participant characteristics for Olink and SomaScan platforms, in subcohort participants only 32

Supplementary Figure 27: Concordance of associations of proteins with participant characteristics between Olink and SomaScan platforms, in subcohort participants only.......... 33

Supplementary Figure 28: Correlation of effect sizes for proteins significantly associated with participant characteristics between Olink and SomaScan platforms, in subcohort participants only.......... 34

Supplementary Figure 29: Number of proteins significantly associated with incident IHD and their effect sizes after applying FDR correction for multiple testing.......... 35

Supplementary Figure 30: Number of proteins significantly associated with incident IHD and their effect sizes after applying Bonferroni correction for multiple testing... 36

Supplementary Figure 31: Comparison of effect sizes for proteins associated with risk of incident IHD between Olink and SomaScan 37

Supplementary Figure 32: Observational correlations and shared associations for IHD 38

Supplementary Figure 33: Factors explaining discordant findings on IHD associations between Olink and SomaScan 39

Supplementary Figure 34: Performance of proteins measured using Olink and SomaScan platforms for prediction of incident IHD using the decile-based method 40
Supplementary Figure 35: Performance of proteins measured using Olink and SomaScan platforms for prediction of incident IHD using the category-free method 41
Supplementary Figure 36: Correlations between protein levels measured by SOMAmers targeting the same protein 42
Supplementary Figure 37: Correlations between protein levels measured by Olink-SomaScan reagent pairs involving multiple SOMAmers 43
Supplementary Figure 38: Correlations between protein levels measured by Olink reagents targeted by the same SOMAmer 44
Supplementary Figure 39: Correlations between protein levels measured by Olink-SomaScan reagent pairs involving multiple Olink reagents 45
Supplementary Figure 40: Consistency of 1,694 one-to-one matched proteins between Olink and SomaScan based on four criteria 46
Supplementary Figure 41: An example of colocalisation of *cis*-pQTLs between a SOMAmer targeting TNC and the corresponding Olink reagent 47
Supplementary Figure 42: An example of no colocalisation of *cis*-pQTLs between a SOMAmer targeting TNC and the corresponding Olink reagent 48
Supplementary Table 1: Protein annotations retrieved from UniProt Knowledgebase for 1,694 overlapping proteins 49
Supplementary Table 2: Top ten most annotated terms under each Gene Ontology (GO) category for 1,694 overlapping proteins 50
Supplementary Table 3: Number of proteins by their number of matched SOMAmers and colocalisation results (ANML) 51
Supplementary Table 4: Number of proteins by their number of matched SOMAmers and colocalisation results (non-ANML) 52

Supplementary Note 1: Members of CKB Collaborative Group

International Steering Committee: Junshi Chen, Zhengming Chen (PI), Robert Clarke, Rory Collins, Liming Li (PI), Chen Wang, Jun Lv, Richard Peto, Robin Walters.

International Co-ordinating Centre, Oxford: Daniel Avery, Maxim Barnard, Derrick Bennett, Ruth Boxall, Ka Hung Chan, Yiping Chen, Zhengming Chen, Jonathan Clarke, Robert Clarke, Huaidong Du, Ahmed Edris Mohamed, Hannah Fry, Simon Gilbert, Pek Kei Im, Andri Iona, Maria Kakkoura, Christiana Kartsonaki, Hubert Lam, Kuang Lin, James Liu, Mohsen Mazidi, Iona Millwood, Sam Morris, Qunhua Nie, Alfred Pozarickij, Paul Ryder, Saredo Said, Dan Schmidt, Becky Stevens, Iain Turnbull, Robin Walters, Baihan Wang, Lin Wang, Neil Wright, Ling Yang, Xiaoming Yang, Pang Yao.

National Co-ordinating Centre, Beijing: Xiao Han, Can Hou, Qingmei Xia, Chao Liu, Jun Lv, Pei Pei, Dianjanyi Sun, Canqing Yu

10 Regional Co-ordinating Centres:

Guangxi Provincial CDC: Naying Chen, Duo Liu, Zhenzhu Tang. Liuzhou CDC: Ningyu Chen, Qilian Jiang, Jian Lan, Mingqiang Li, Yun Liu, Fanwen Meng, Jinhuai Meng, Rong Pan, Yulu Qin, Ping Wang, Sisi Wang, Liuping Wei, Liyuan Zhou. **Gansu** Provincial CDC: Caixia Dong, Pengfei Ge, Xiaolan Ren. Maiji CDC: Zhongxiao Li, Enke Mao, Tao Wang, Hui Zhang, Xi Zhang. **Hainan** Provincial CDC: Jinyan Chen, Ximin Hu, Xiaohuan Wang. Meilan CDC: Zhendong Guo, Huimei Li, Yilei Li, Min Weng, Shukuan Wu. **Heilongjiang** Provincial CDC: Shichun Yan, Mingyuan Zou, Xue Zhou. Nangang CDC: Ziyan Guo, Quan Kang, Yanjie Li, Bo Yu, Qinai Xu. **Henan** Provincial CDC: Liang Chang, Lei Fan, Shixian Feng, Ding Zhang, Gang Zhou. Huixian CDC: Yulian Gao, Tianyou He, Pan He, Chen Hu, Huarong Sun, Xukui Zhang. **Hunan** Provincial CDC: Biyun Chen, Zhongxi Fu, Yuelong Huang, Huilin Liu, Qiaohua Xu, Li Yin. Liuyang CDC: Huajun Long, Xin Xu, Hao Zhang, Libo Zhang. **Jiangsu** Provincial CDC: Jian Su, Ran Tao, Ming Wu, Jie Yang, Jinyi Zhou, Yonglin Zhou. Suzhou CDC: Yihe Hu, Yujie Hua, Jianrong Jin, Fang Liu, Jingchao Liu, Yan Lu, Liangcai Ma, Aiyu Tang, Jun Zhang. **Qingdao** CDC: Liang Cheng, Ranran Du, Ruqin Gao, Feifei Li, Shanpeng Li, Yongmei Liu, Feng Ning, Zengchang Pang, Xiaohui Sun, Xiaocao Tian, Shaojie Wang, Yaoming Zhai, Hua Zhang, Licang CDC: Wei Hou, Silu Lv, Junzheng Wang. **Sichuan** Provincial CDC: Xiaofang Chen, Xianping Wu, Ningmei Zhang, Weiwei Zhou. Pengzhou CDC: Xiaofang Chen, Jianguo Li, Jiaqiu Liu, Guojin Luo, Qiang Sun, Xunfu Zhong. **Zhejiang** Provincial CDC: Weiwei Gong, Ruying Hu, Hao Wang, Meng Wang, Min Yu. Tongxiang CDC: Lingli Chen, Qijun Gu, Dongxia Pan, Chunmei Wang, Kaixu Xie, Xiaoyi Zhang.

Supplementary Note 2: Reagent matching based on observational correlations

To check the validity of our reagent matching procedure based on UniProt IDs, we calculated the correlations of all possible reagent pairs between Olink and SomaScan for the 2,168 proteins included in the analysis.

As shown in the heatmap (Supplementary Figure 6), although the correlations seem randomly distributed overall, a faint diagonal line representing the UniProt-matched pairs is still visible. This is consistent with our finding of the modest correlation between the UniProt-matched pairs with a median rho = 0.23 (Olink vs SomaScan-ANML) / 0.29 (Olink vs SomaScan-non-ANML).

Of the 2,168 proteins, 739 pairs were mutual best hits between Olink and SomaScan-ANML (where the reagents were each other's best hits) and 739 were mutual best hits between Olink and SomaScan-non-ANML. This could be a result of reagents binding different proteoforms of the same protein, or could indicate the binding of reagents on non-target proteins. However, 701 of the 739 mutual best hits between Olink and SomaScan-ANML were also matches based on UniProt IDs, while 706 of the 739 mutual best hits between Olink and SomaScan-non-ANML were also matches based on UniProt IDs. This suggests that our matching method based on UniProt IDs is a valid approach.

Supplementary Note 3: Effect of ambient temperature and time since last meal on protein levels

To investigate the influence of ambient temperature and time since last meal on protein levels measured by Olink and SomaScan, we performed a principal component analysis (PCA) on all 1,694 one-to-one matched proteins in each platform. The scree plots showing the variances explained by the top 10 PCs were shown in Supplementary Figure 19.

We then tested the associations between ambient temperature/time since last meal and the first PC of each platform. We performed using linear regression with PC1 as the outcome, while adjusting for other covariates (age, age^2, sex, and plate ID). We found a significant association between PC1 of SomaScan-ANML and ambient temperature ($p < 0.001$), while PC1 of Olink, SomaScan-ANML, and SomaScan-non-ANML were all significantly associated with time since last meal ($ps < 0.01$). This suggests the general influence of environmental/technical factors on proteomic measurements. We also observed a number of proteins associated with ambient temperature and time since last meal in both platforms, as shown in Supplementary Figures 20 to 25.

Supplementary Figure 1: Correlations of levels of 2,168 proteins (2,749 pairs) between Olink and SomaScan

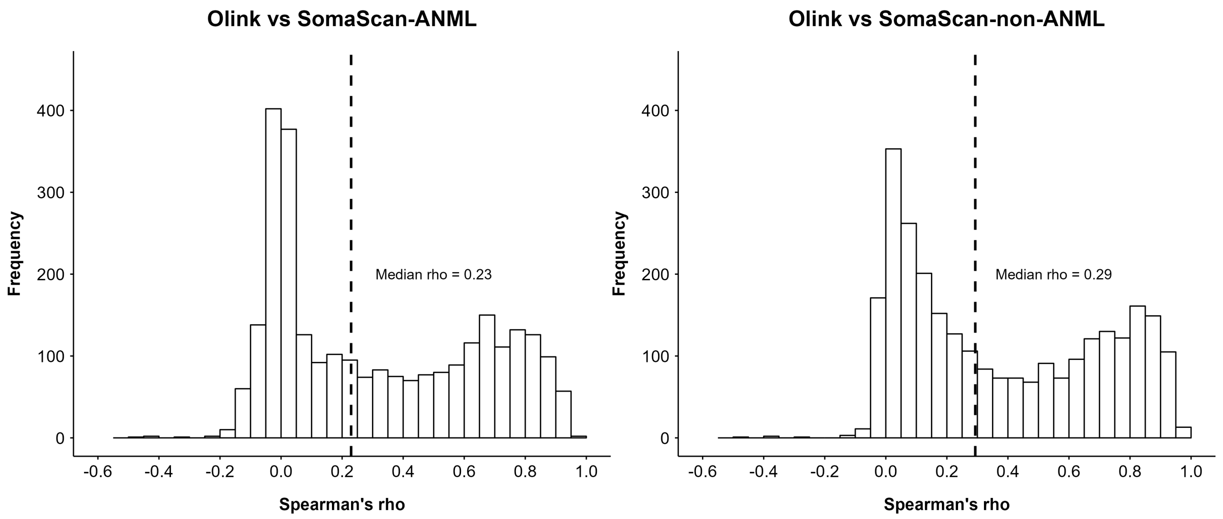

Supplementary Figure 2: Pearson's r of levels of 2,168 proteins (2,749 pairs) between Olink and SomaScan

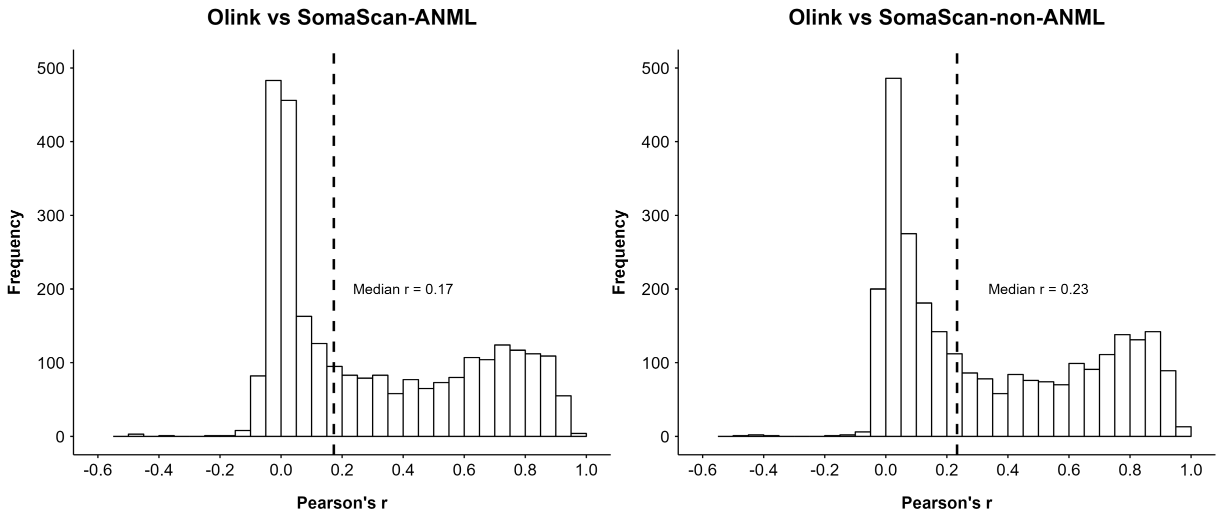

Supplementary Figure 3: Correlations of levels of 1,694 one-to-one matched proteins between Olink and SomaScan

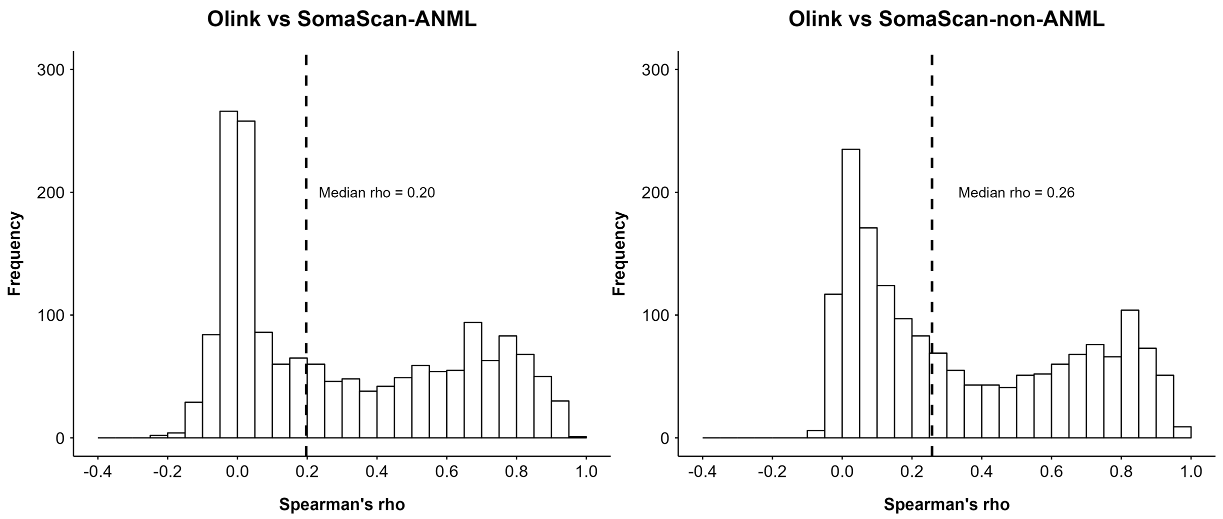

Supplementary Figure 4: Pearson's r of levels of 1,694 proteins between Olink and SomaScan

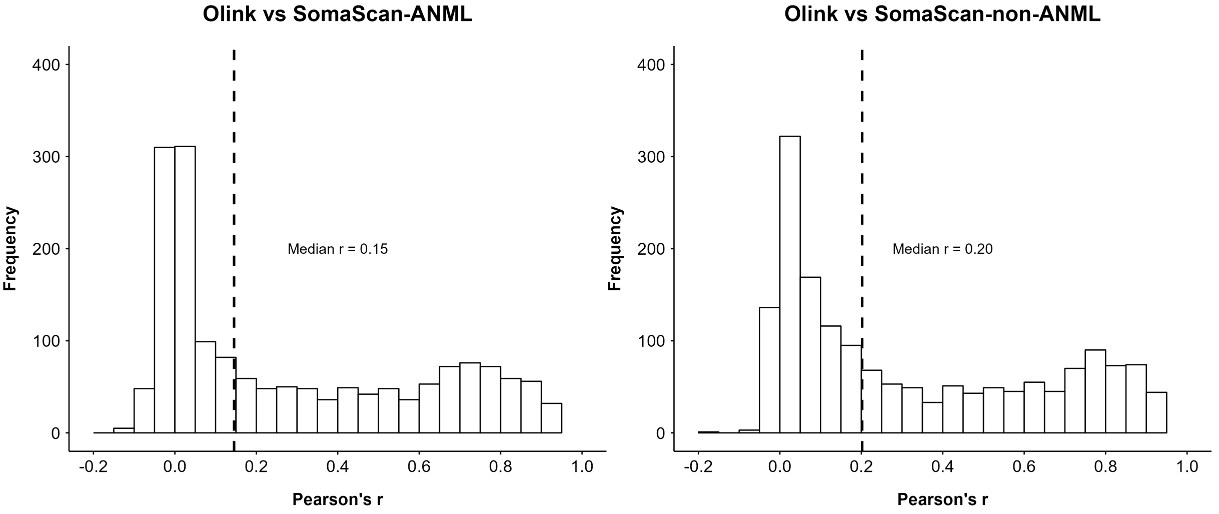

Supplementary Figure 5: Correlations of levels of 1,694 proteins between Olink and SomaScan in 2,025 subcohort participants

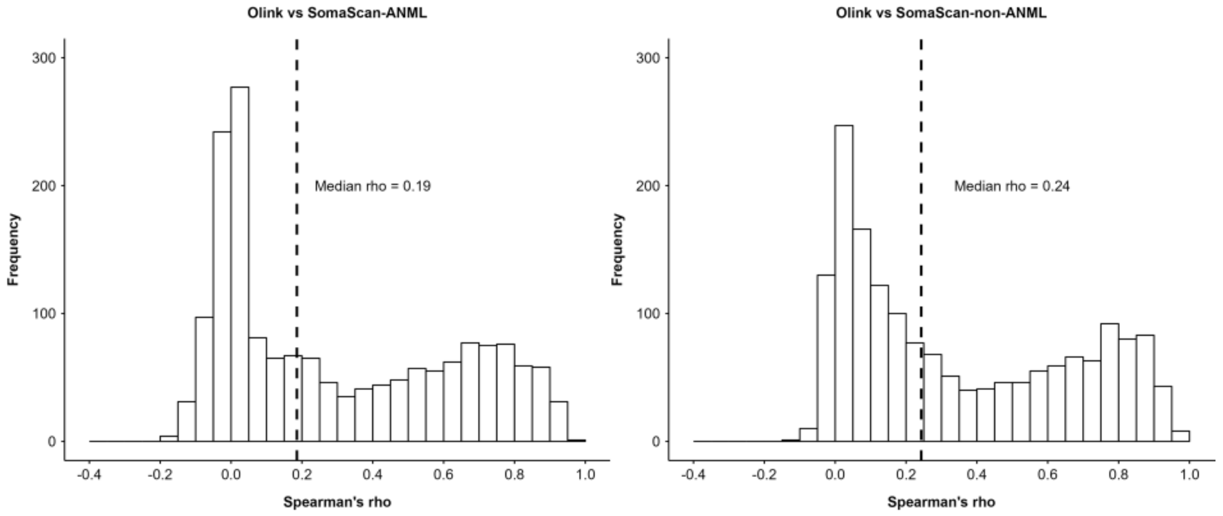

Supplementary Figure 6: Spearman’s rho of all possible regent pairs between Olink and SomaScan for 2,168 proteins

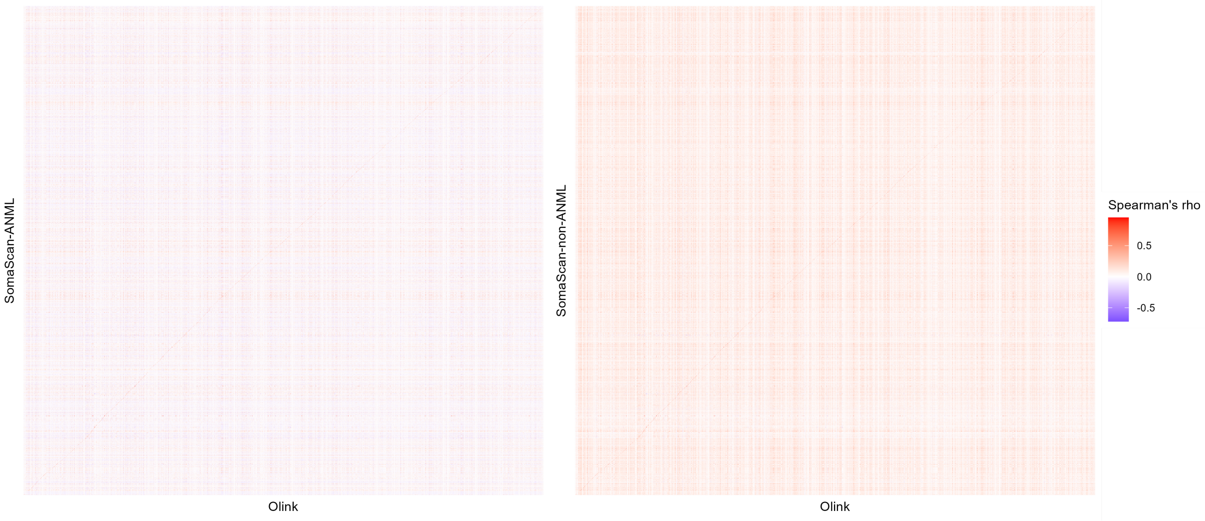

Supplementary Figure 7: Features predictive of correlations in Boruta feature selection

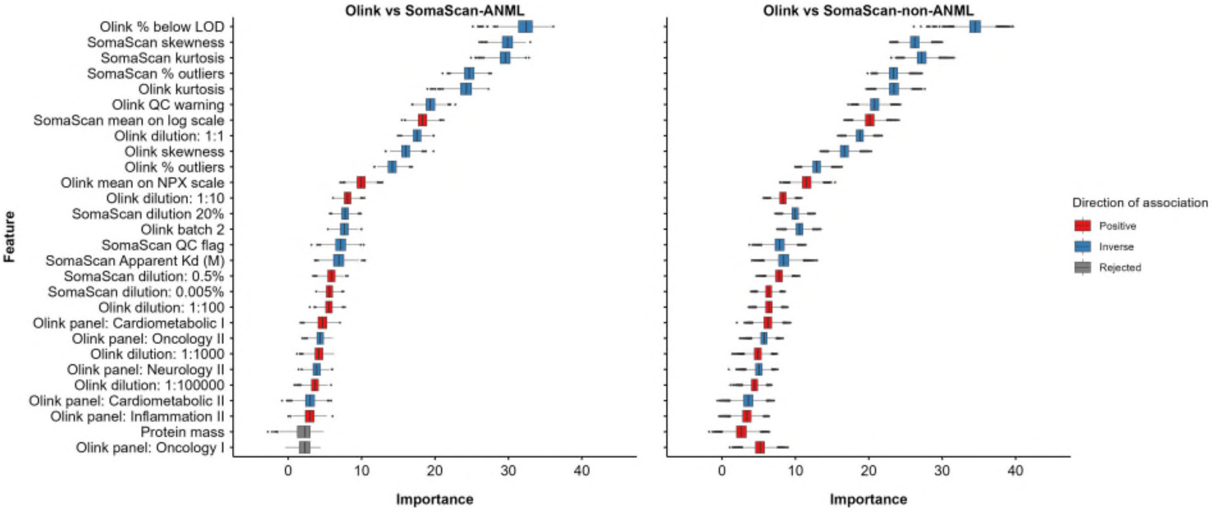

Supplementary Figure 8: Correlations between protein levels measured by Olink and SomaScan platforms according to degree of dilution in Olink assay

Dashed lines indicate the median rho.

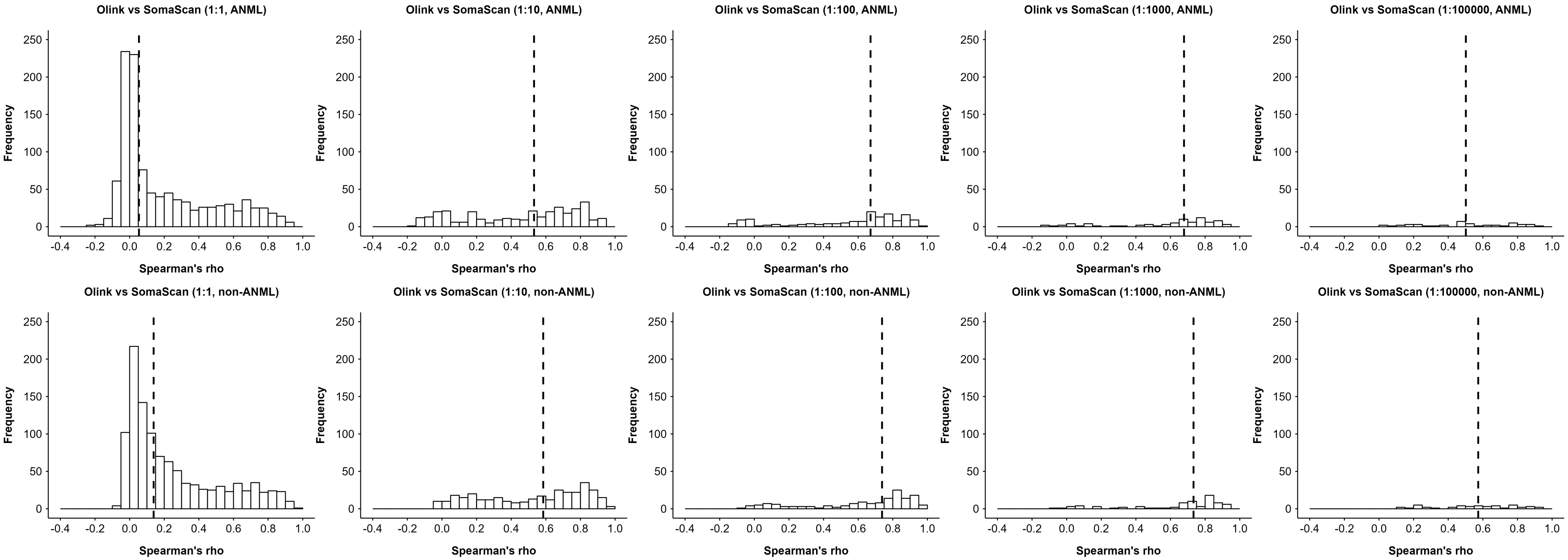

Supplementary Figure 9: Correlations between protein levels measured by Olink and SomaScan platforms according to degree of dilution in SomaScan assay

Dashed lines indicate the median rho.

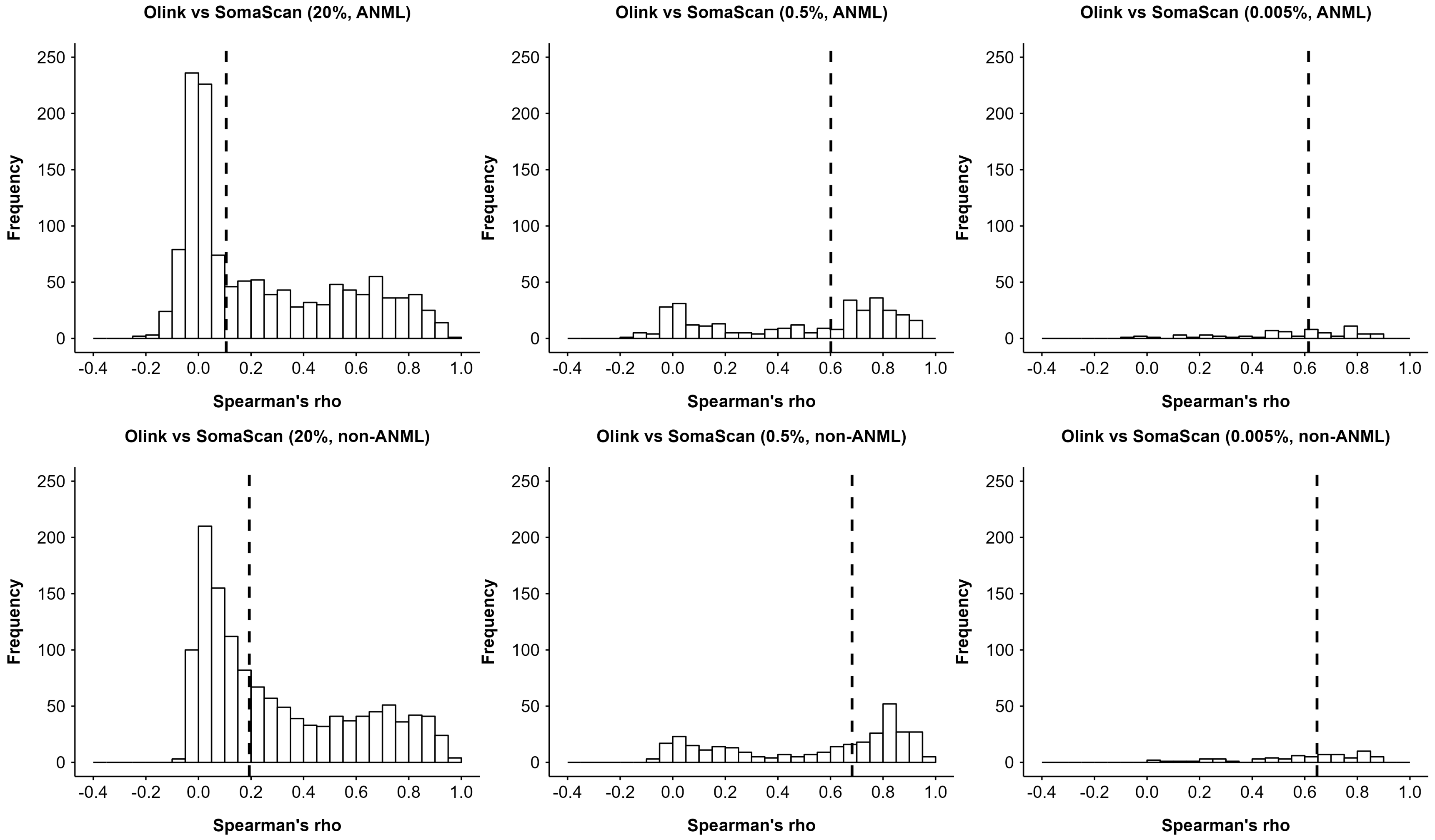

Supplementary Figure 10: number of pQTLs identified in each platform.

Number of sentinel pQTLs identified in each platform, with shaded areas indicating the number of proteins with pQTLs.

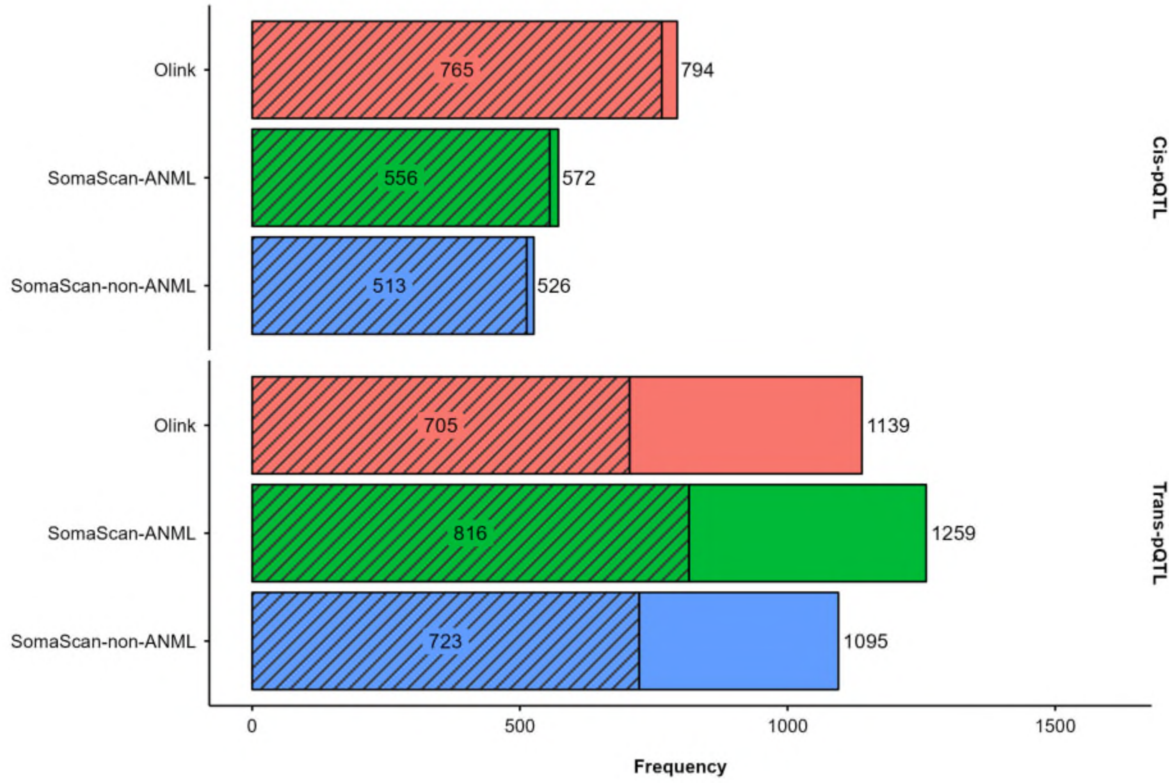

Supplementary Figure 11: Number of proteins with *cis*-pQTLs discovered in Olink and SomaScan and colocalisation results

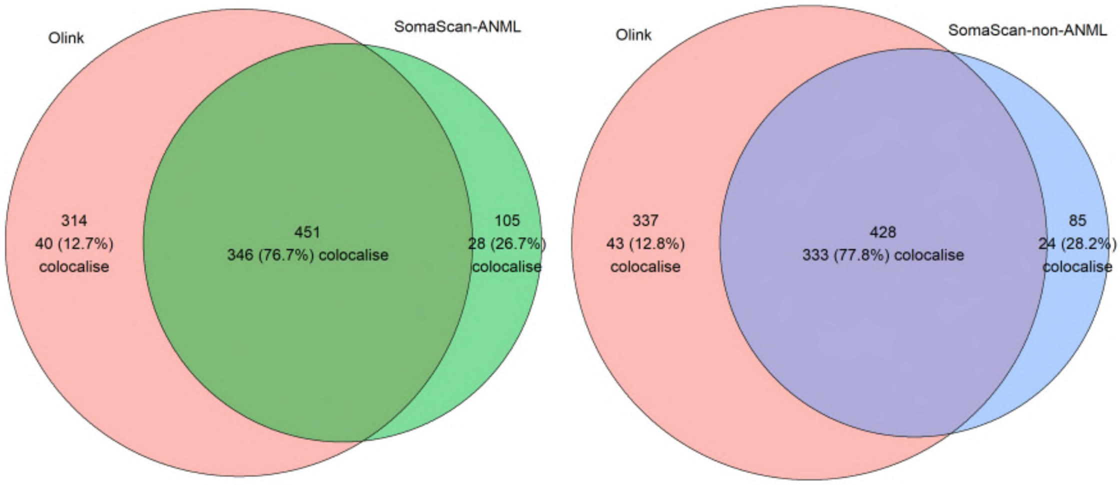

Supplementary Figure 12: Observational correlations between Olink and SomaScan and proteins with colocalising *cis*-pQTLs

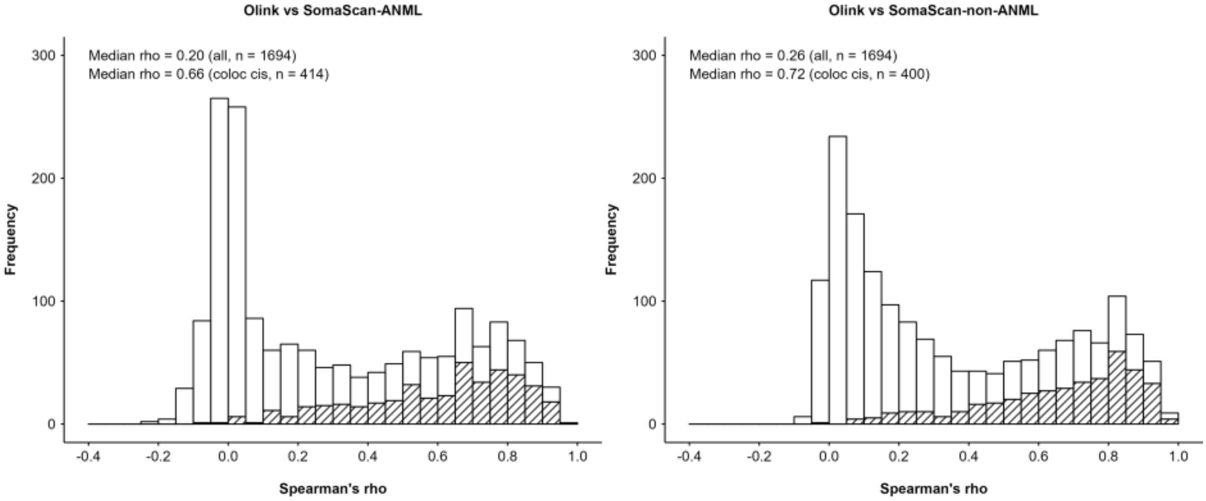

Supplementary Figure 13: Factors explaining discordant findings on *cis*-pQTLs between Olink and SomaScan

Results were shown based on SomaScan-ANML (A) and SomaScan-non-ANML (B). Proteins were categorised into four groups: proteins with only Olink-specific *cis*-pQTLs, proteins with only SomaScan-specific *cis*-pQTL, proteins with *cis*-pQTLs in both platforms which did not colocalise, and proteins with colocalising *cis*-pQTLs between platforms. Using proteins with colocalising *cis*-pQTLs as the reference group, we tested if observational correlations, key technical factors, or protein-altering variants were could explain discordant findings on *cis*-pQTLs between platforms. Betas represent effect sizes per SD change for continuous factors, or log odds ratios for binary variables. *: Continuous factors were scaled to show comparable effect sizes in the figures; LOD=limit of detection; QC=quality control; PAV=protein-altering variants.

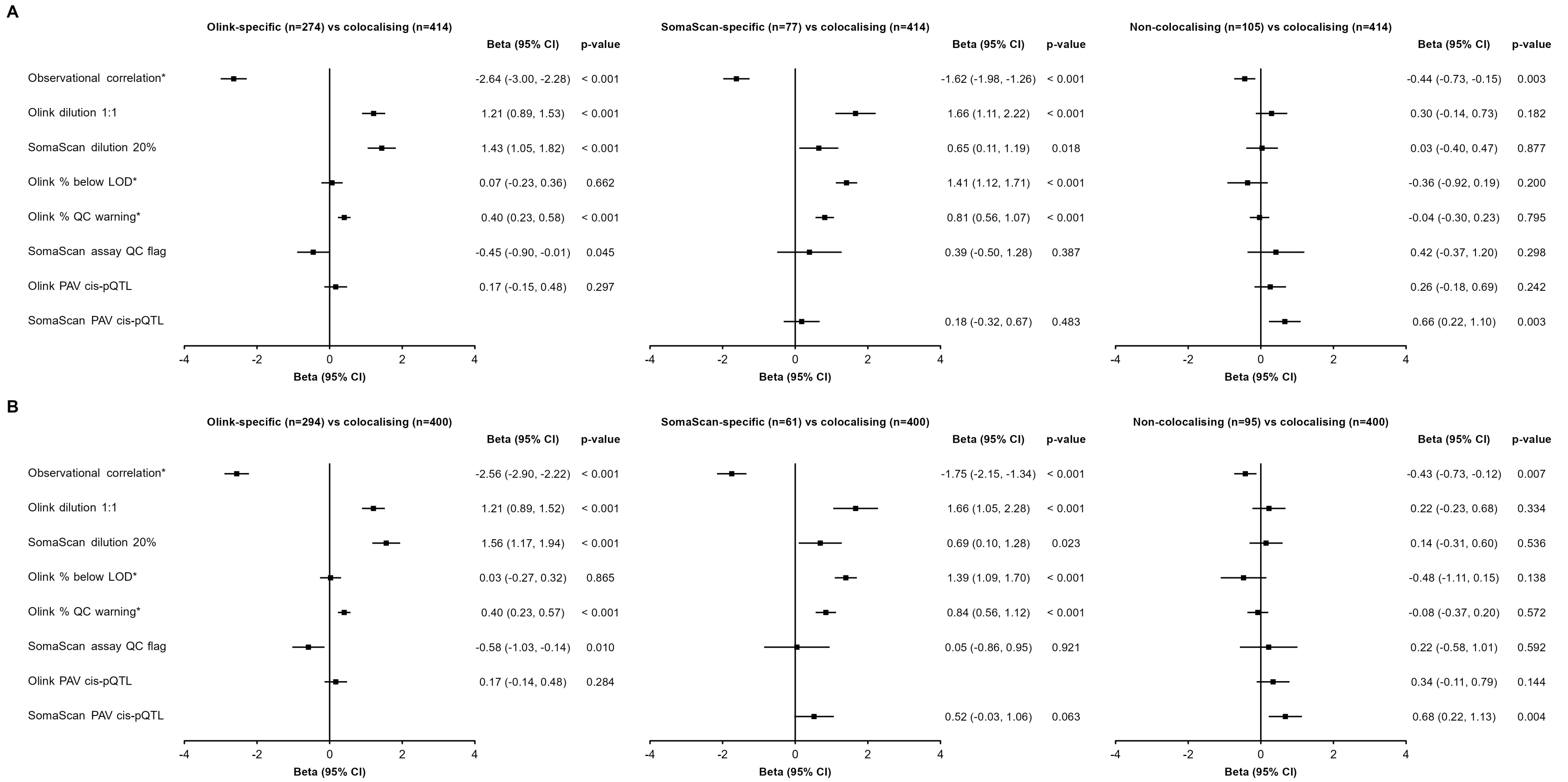

Supplementary Figure 14: *cis*-pQTLs for ALDH2 identified in Olink and SomaScan platforms and colocalisation results

SomaScan results were based on the non-ANML data. MAF=Minor allele frequency; PP=Posterior probability from colocalisation. Detailed information on *cis*-pQTLs for ALDH2 is shown in Supplementary Data 4.

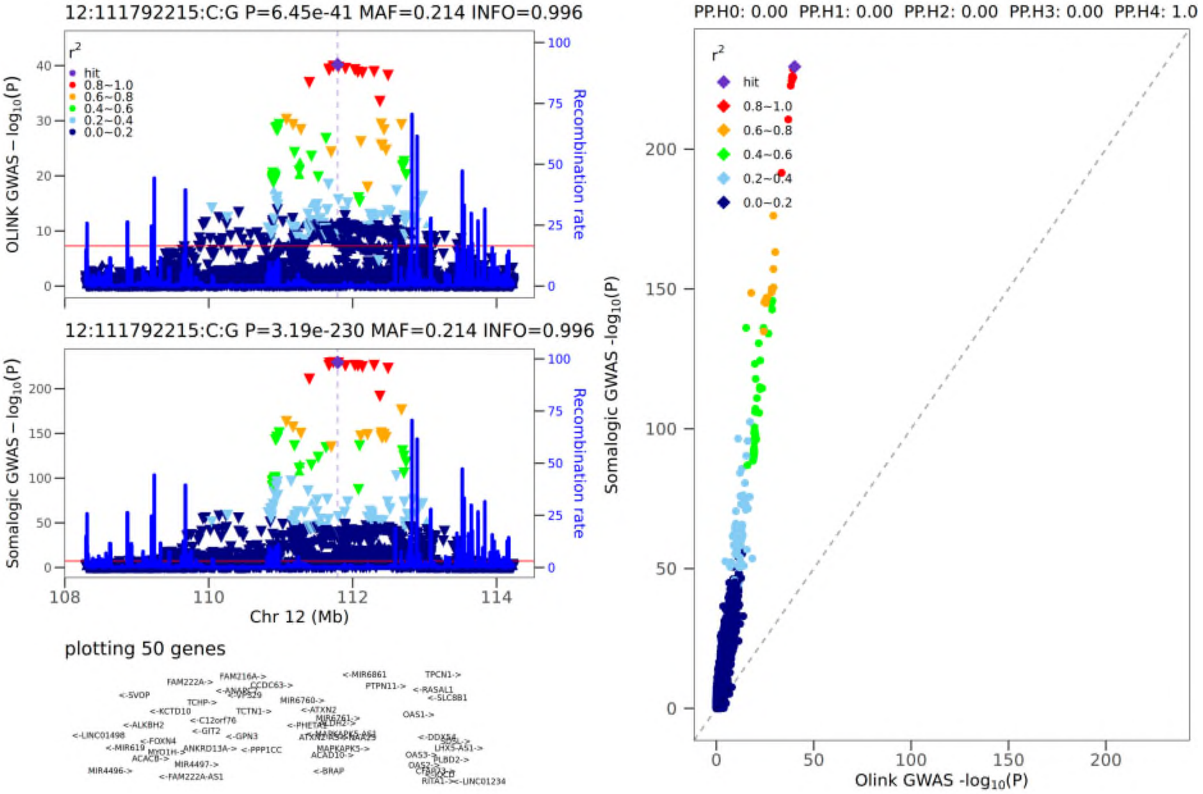

Supplementary Figure 15: *cis*-pQTLs for PLA2G7 identified in Olink and SomaScan platforms and colocalisation results

SomaScan results were based on the non-ANML data. MAF=Minor allele frequency; PP=Posterior probability from colocalisation. Detailed information on *cis*-pQTLs for PLA2G7 is shown in Supplementary Data 4.

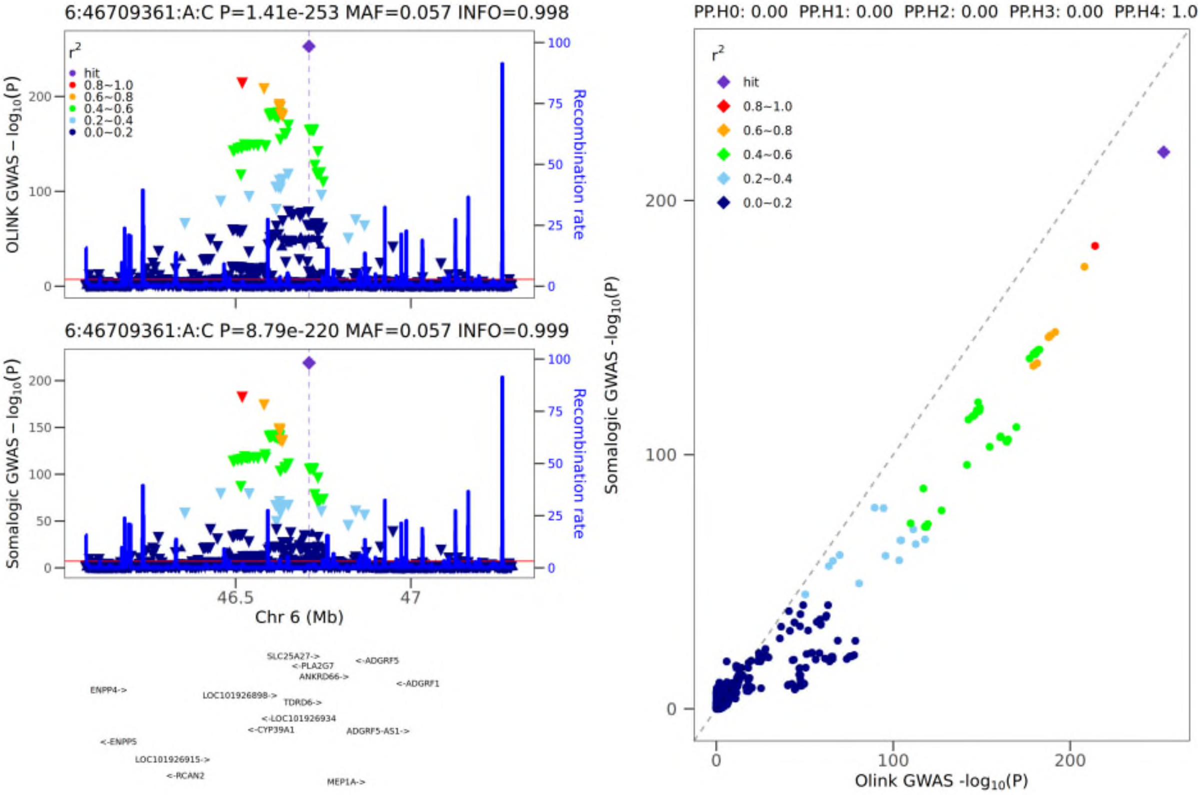

Supplementary Figure 16: *cis*-pQTLs for PCSK9 identified in Olink and SomaScan platforms and colocalisation results

SomaScan results were based on the non-ANML data. MAF=Minor allele frequency; PP=Posterior probability from colocalisation. Detailed information on *cis*-pQTLs for PCSK9 is shown in Supplementary Data 4.

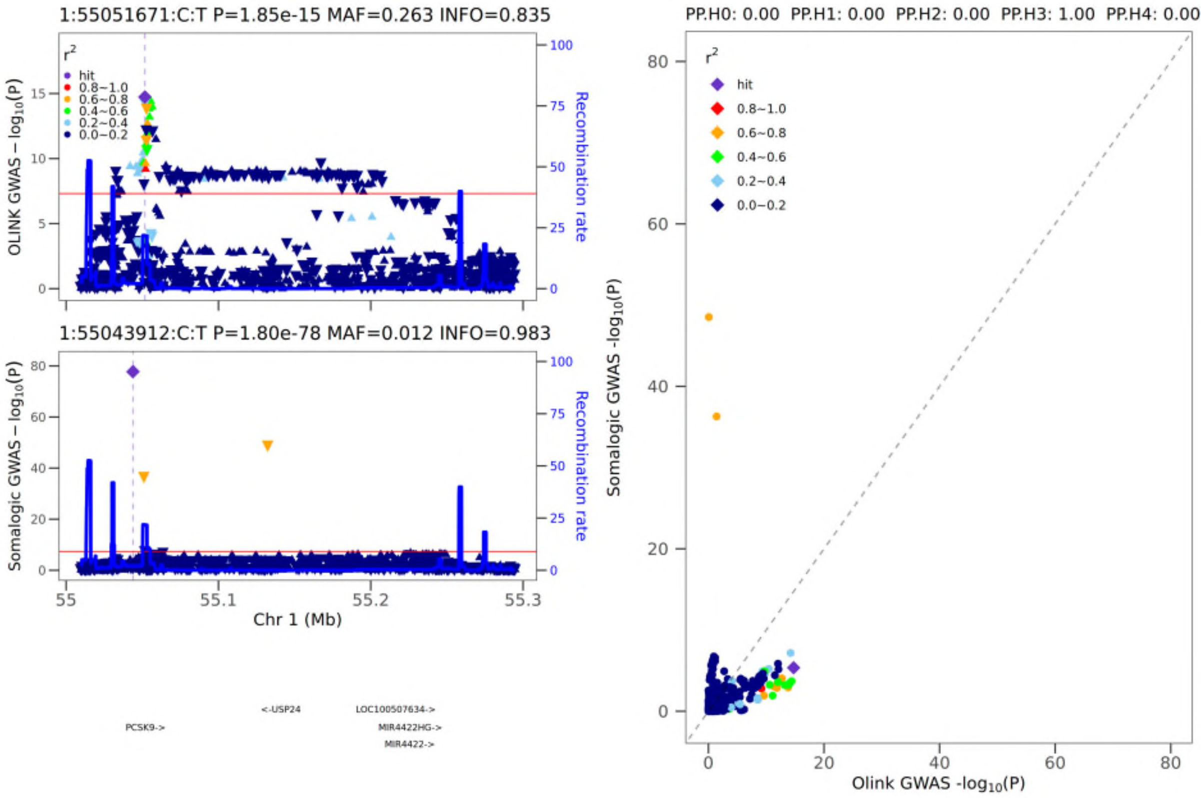

Supplementary Figure 17: Number of proteins significantly associated with BMI and their effect sizes after applying FDR correction for multiple testing

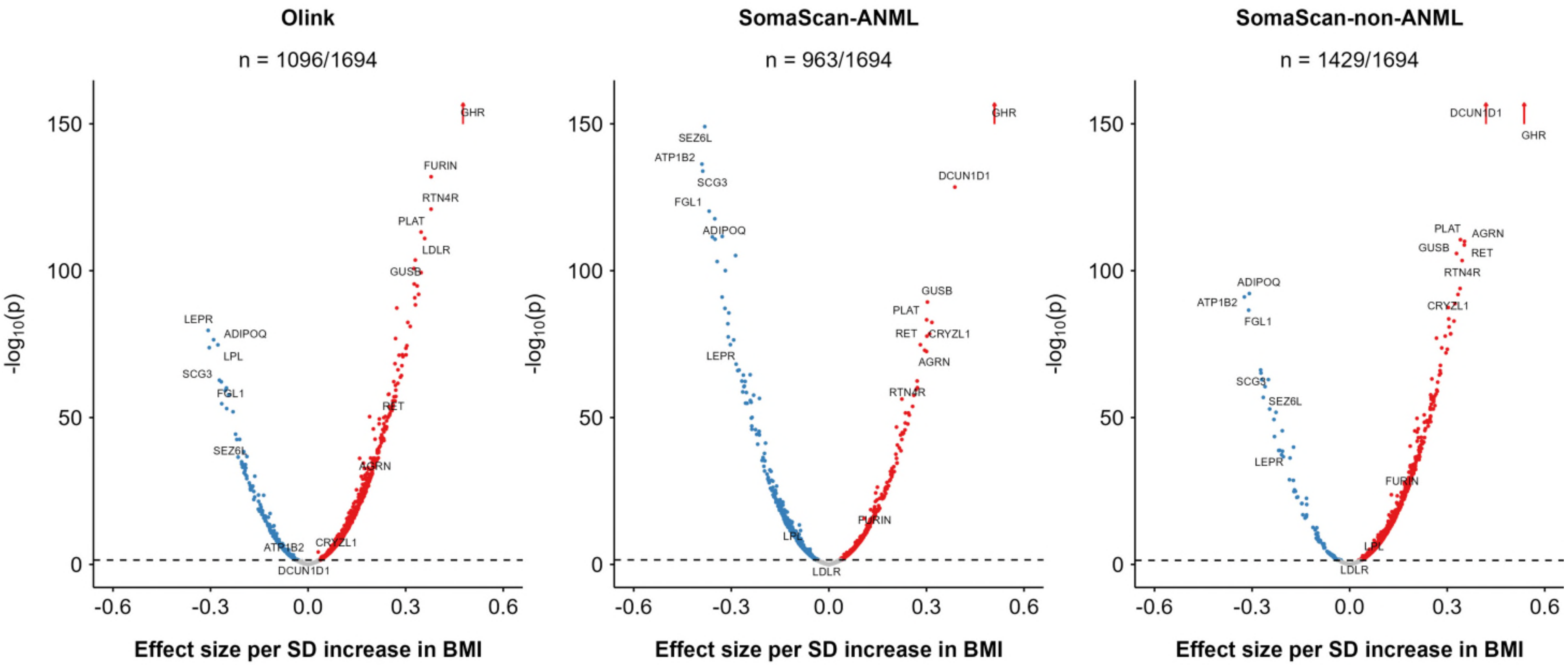

Supplementary Figure 18: Number of proteins significantly associated with BMI and their effect sizes after applying Bonferroni correction for multiple testing

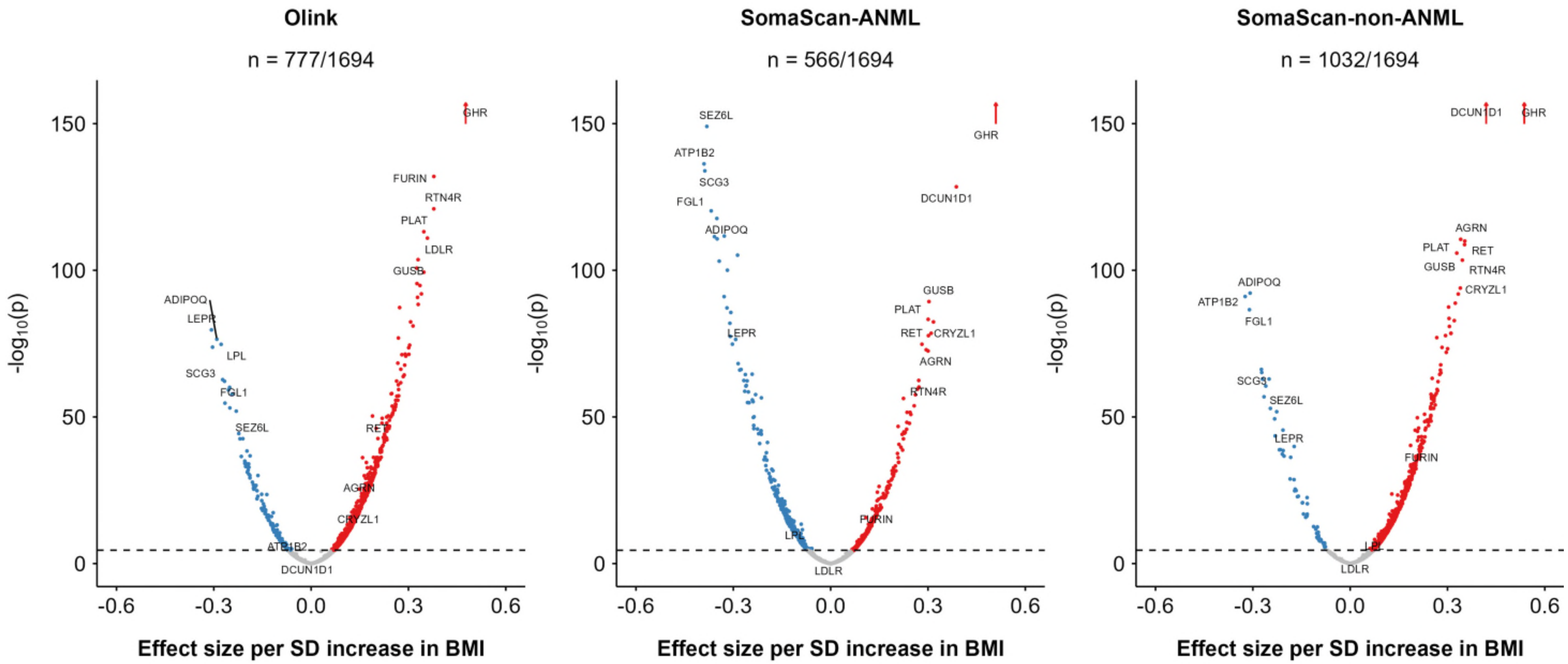

Supplementary Figure 19: Comparison of effect sizes for proteins associated with BMI between Olink and SomaScan

Dark dots indicate shared associations between Olink and SomaScan, which were defined as significant associations found in both datasets that were also directionally consistent. Results were corrected using false discovery rate.

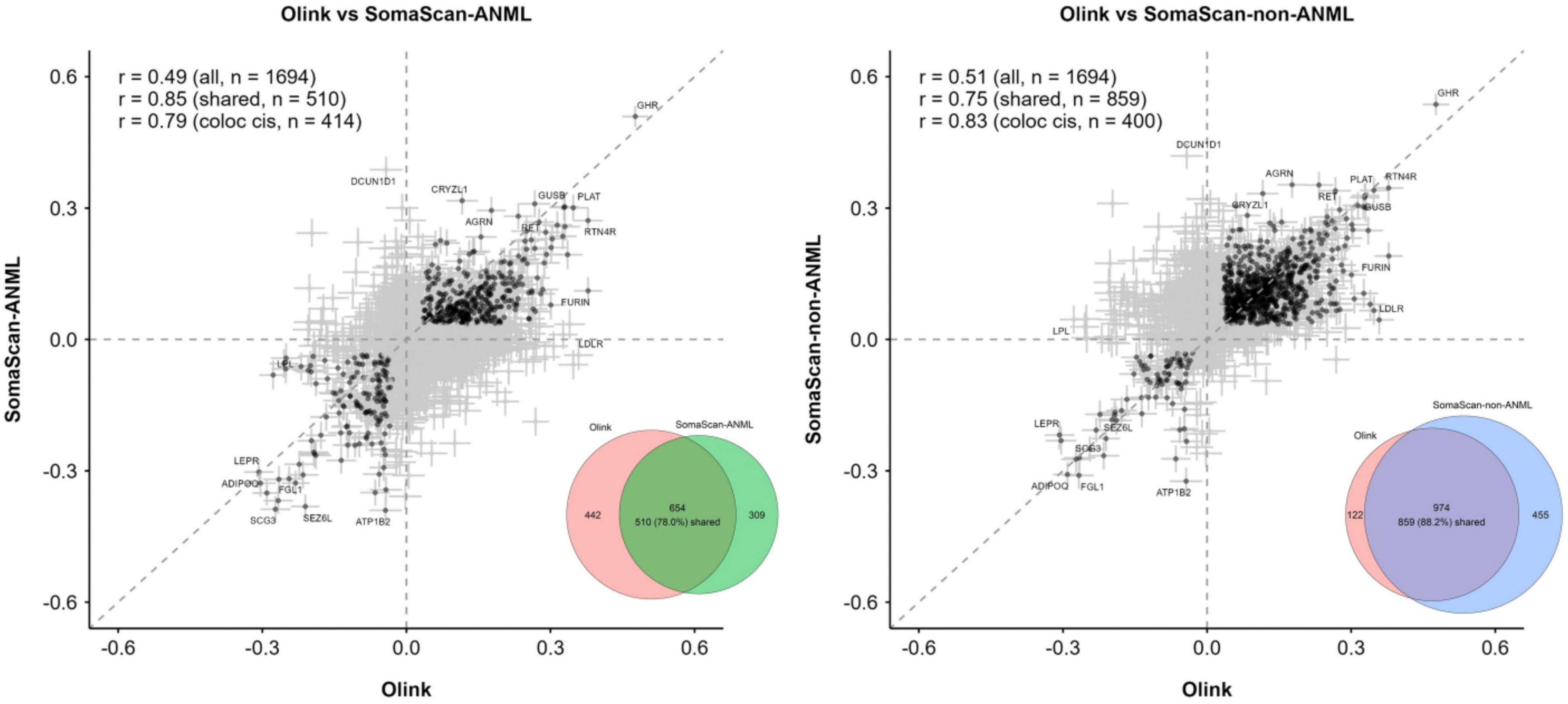

Supplementary Figure 20: Observational correlations and shared associations for BMI

Results were corrected for multiple testing using false discovery rate within each platform.

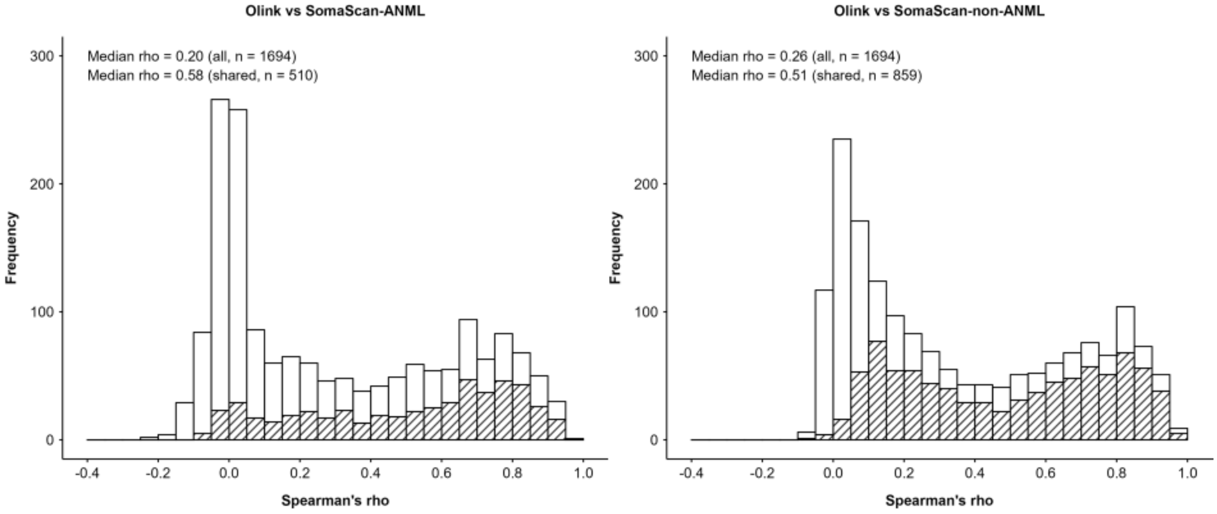

Supplementary Figure 21: Factors explaining discordant findings on BMI associations between Olink and SomaScan

Results were shown based on SomaScan-ANML (A) and SomaScan-non-ANML (B). Proteins were categorised into four groups: proteins associated with BMI in Olink only, proteins associated with BMI in SomaScan only, proteins associated with BMI in both platforms but with different directions of their effects, and proteins associated with BMI in both platforms and with the same direction of their effects (shared). Using shared proteins as the reference group, we tested if observational correlations, key technical factors, or protein-altering variants were could explain discordant findings on BMI associations between platforms. Betas represent effect sizes per SD change for continuous factors, or log odds ratios for binary variables. *: Continuous factors were scaled to show comparable effect sizes in the figures; LOD=limit of detection; QC=quality control; PAV=protein-altering variants.

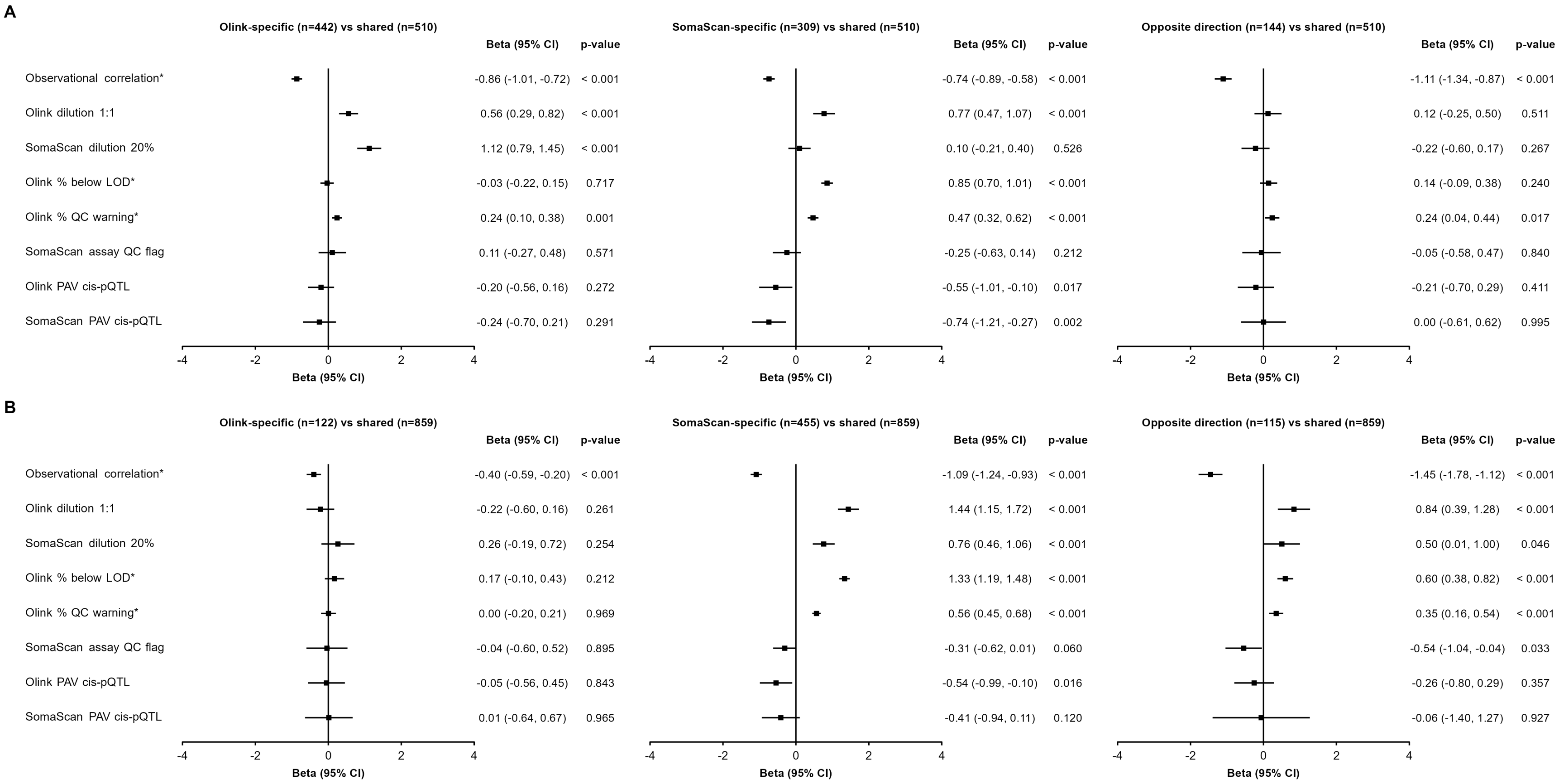

Supplementary Figure 22: Variance explained by top 10 principal components in each platform

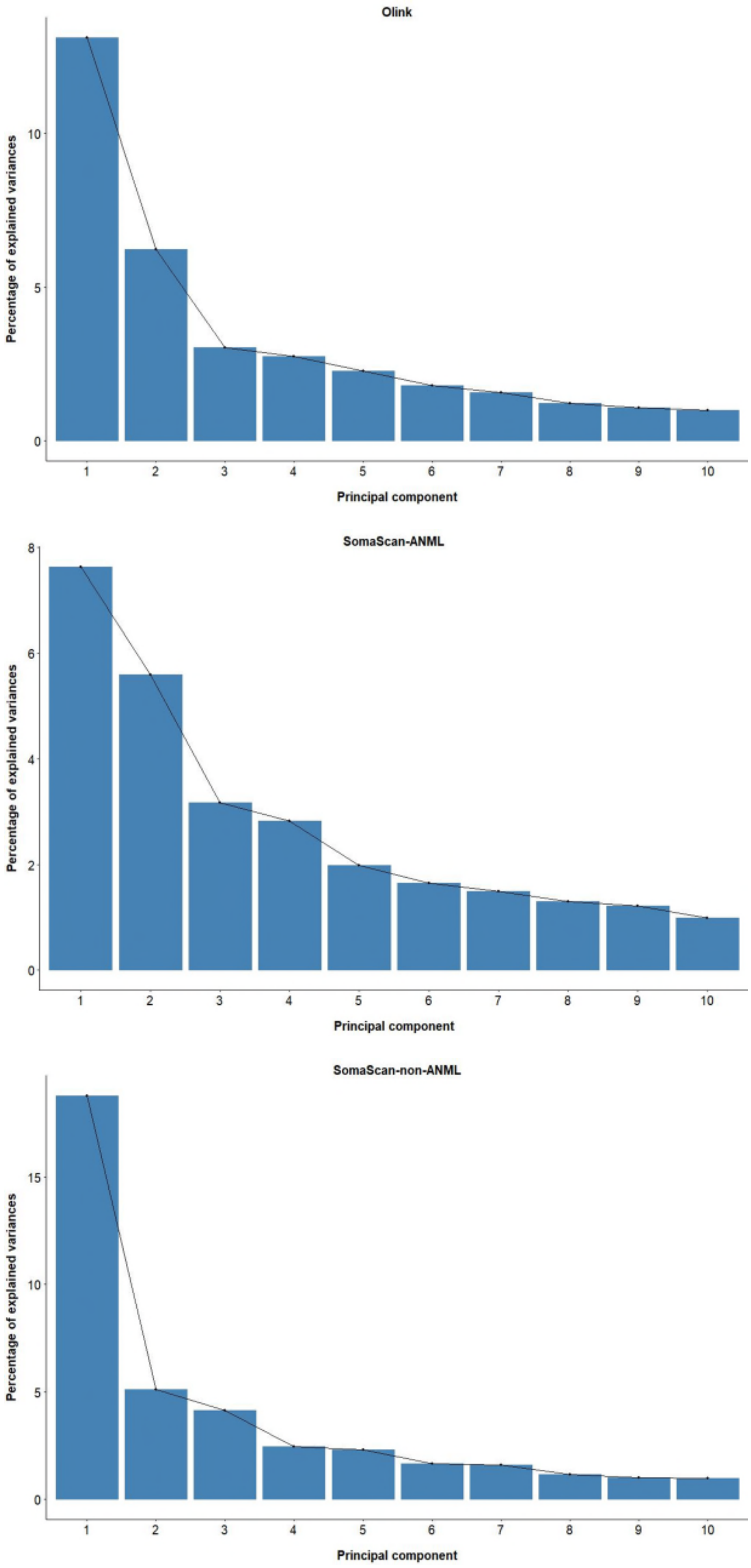

Supplementary Figure 23: Number of proteins significantly associated with selected baseline characteristics for Olink and SomaScan platforms

Results were corrected using false discovery rate within each trait and each platform. Analyses on ever regular smoking and regular alcohol drinking were conducted in male participants only.

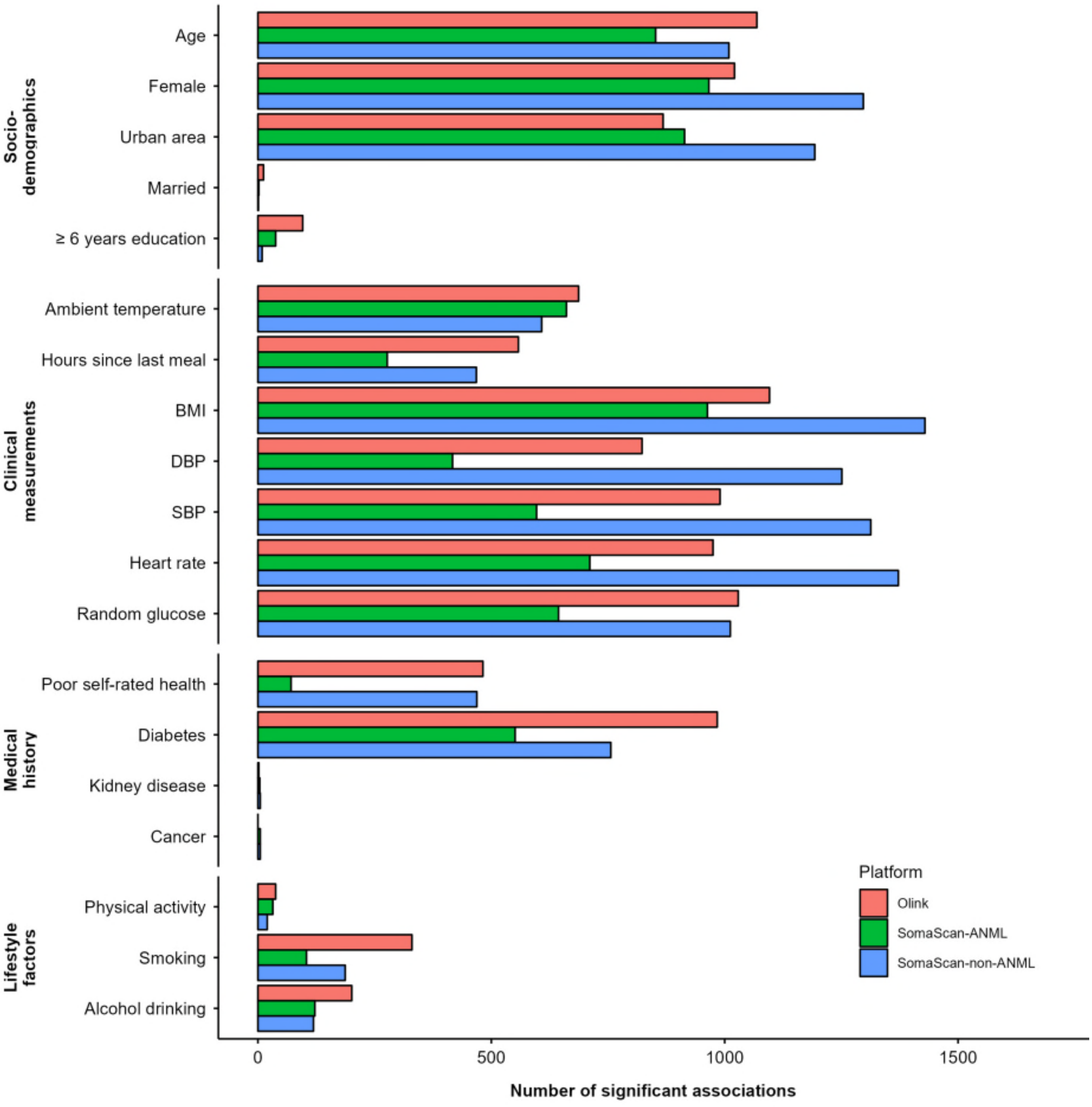

Supplementary Figure 24: Concordance of associations of proteins with participant characteristics between Olink and SomaScan platforms

Results were corrected using false discovery rate within each trait and each platform. Shared associations were defined as significant associations found in both platforms that were also directionally consistent. Results were corrected using false discovery rate. Analyses on ever regular smoking and regular alcohol drinking were conducted in male participants only.

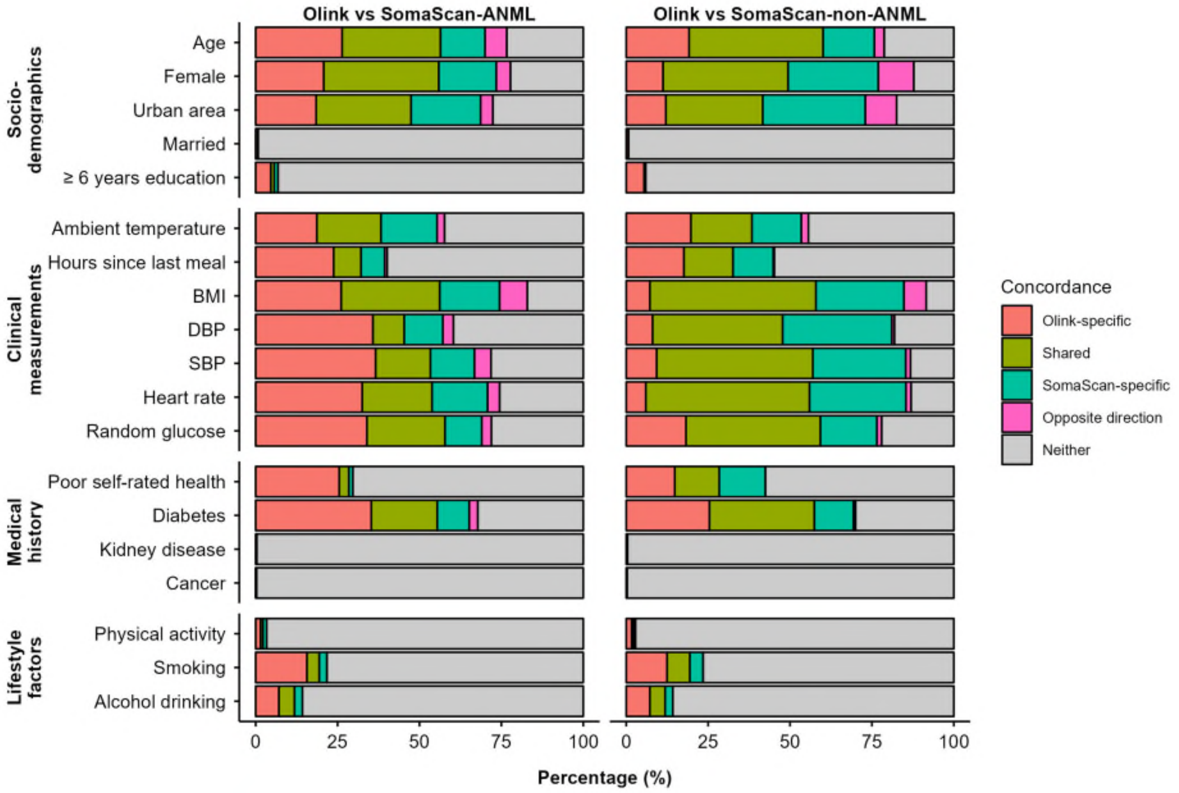

Supplementary Figure 25: Correlation of effect sizes for proteins significantly associated with participant characteristics between Olink and SomaScan platforms

Results were corrected using false discovery rate within each trait and each platform. Shared associations were defined as significant associations found in both platforms that were also directionally consistent. Analyses on ever regular smoking and regular alcohol drinking were conducted in male participants only.

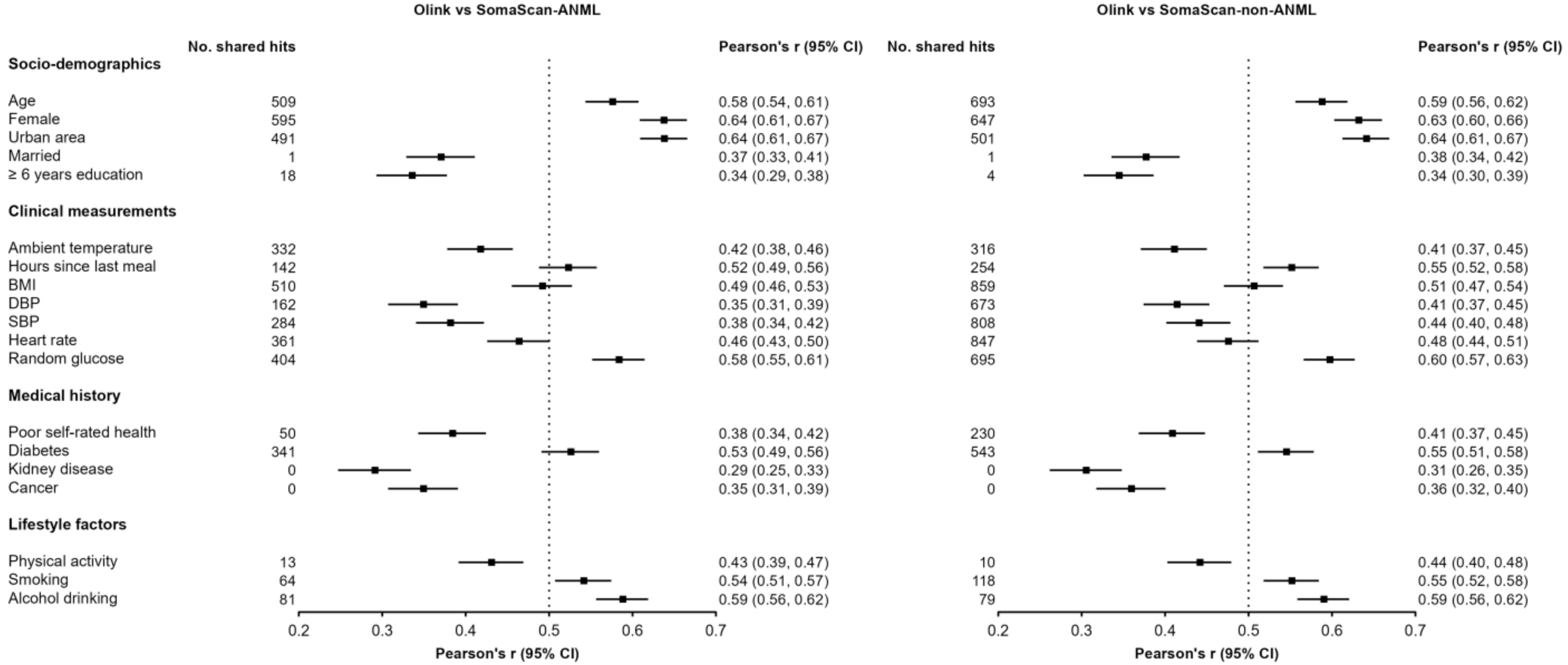

Supplementary Figure 26: Number of proteins significantly associated with participant characteristics for Olink and SomaScan platforms, in subcohort participants only

Results were corrected using false discovery rate within each trait and each platform. Analyses on ever regular smoking and regular alcohol drinking were conducted in male participants only.

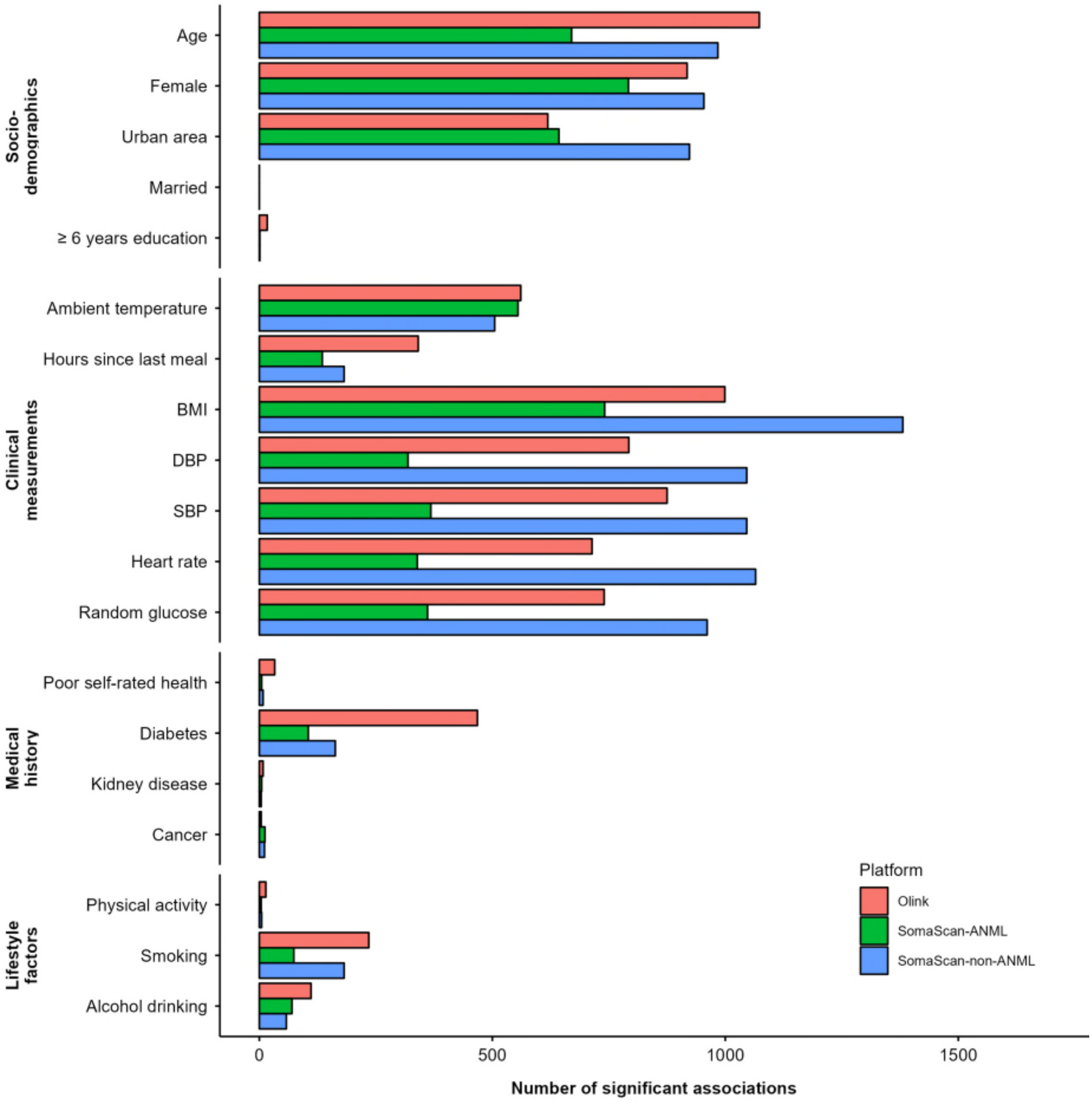

Supplementary Figure 27: Concordance of associations of proteins with participant characteristics between Olink and SomaScan platforms, in subcohort participants only

Results were corrected using false discovery rate within each trait and each platform. Shared associations were defined as significant associations found in both platforms that were also directionally consistent. Analyses on ever regular smoking and regular alcohol drinking were conducted in male participants only.

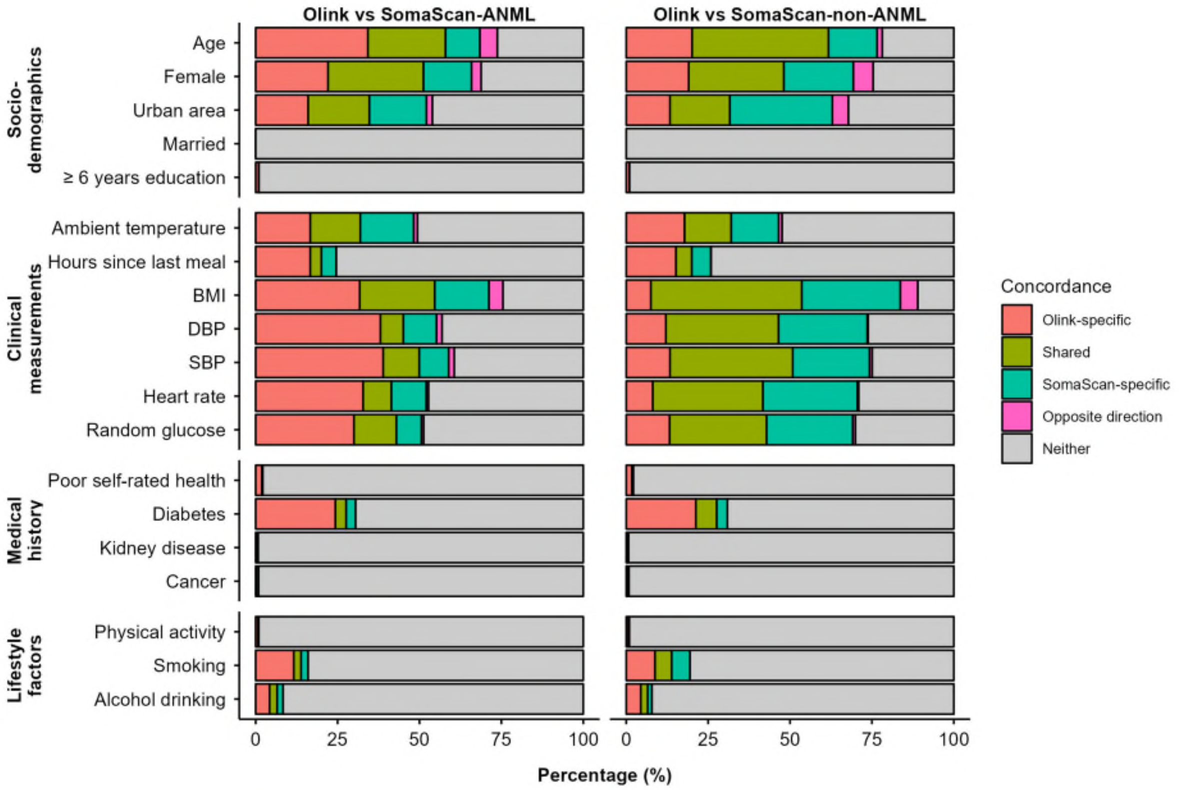

Supplementary Figure 28: Correlation of effect sizes for proteins significantly associated with participant characteristics between Olink and SomaScan platforms, in subcohort participants only

Results were corrected using false discovery rate within each trait and each platform. Shared associations were defined as significant associations found in both platforms that were also directionally consistent. Analyses on ever regular smoking and regular alcohol drinking were conducted in male participants only.

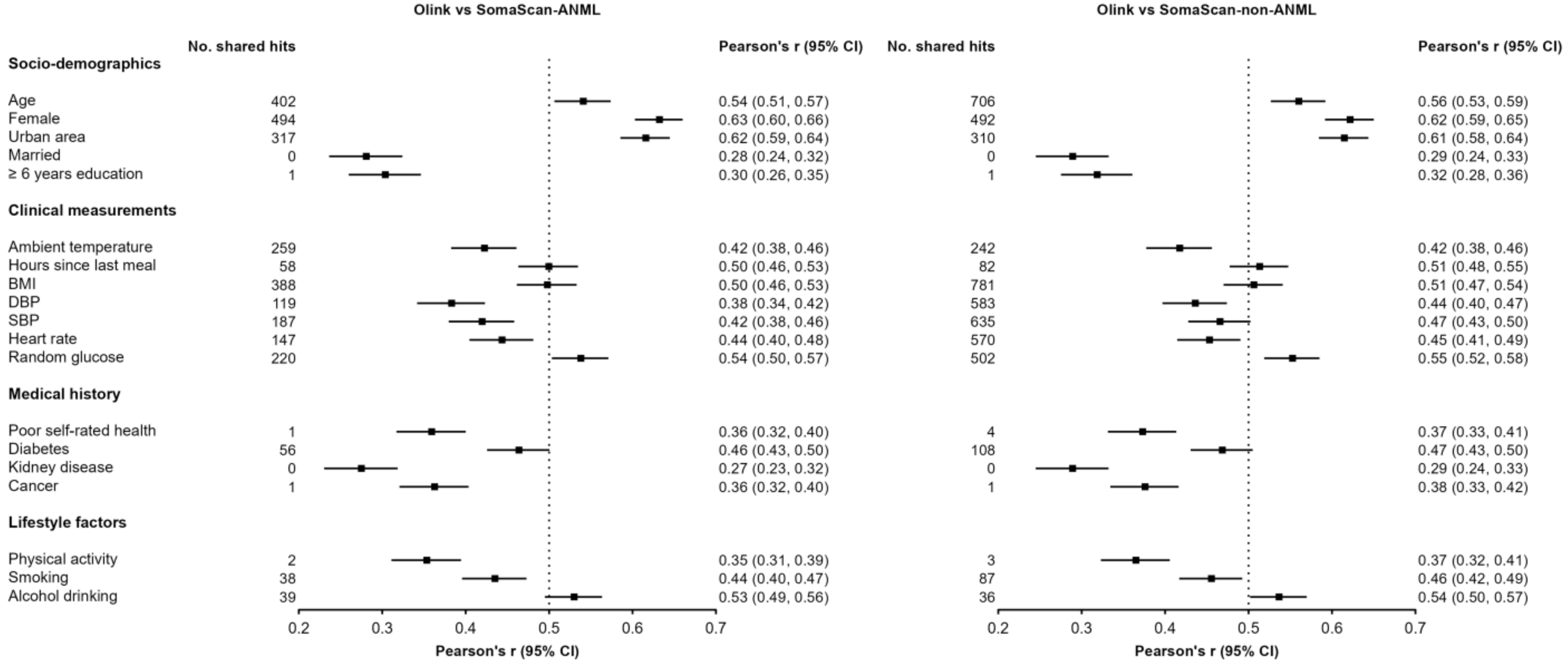

Supplementary Figure 29: Number of proteins significantly associated with incident IHD and their effect sizes after applying FDR correction for multiple testing

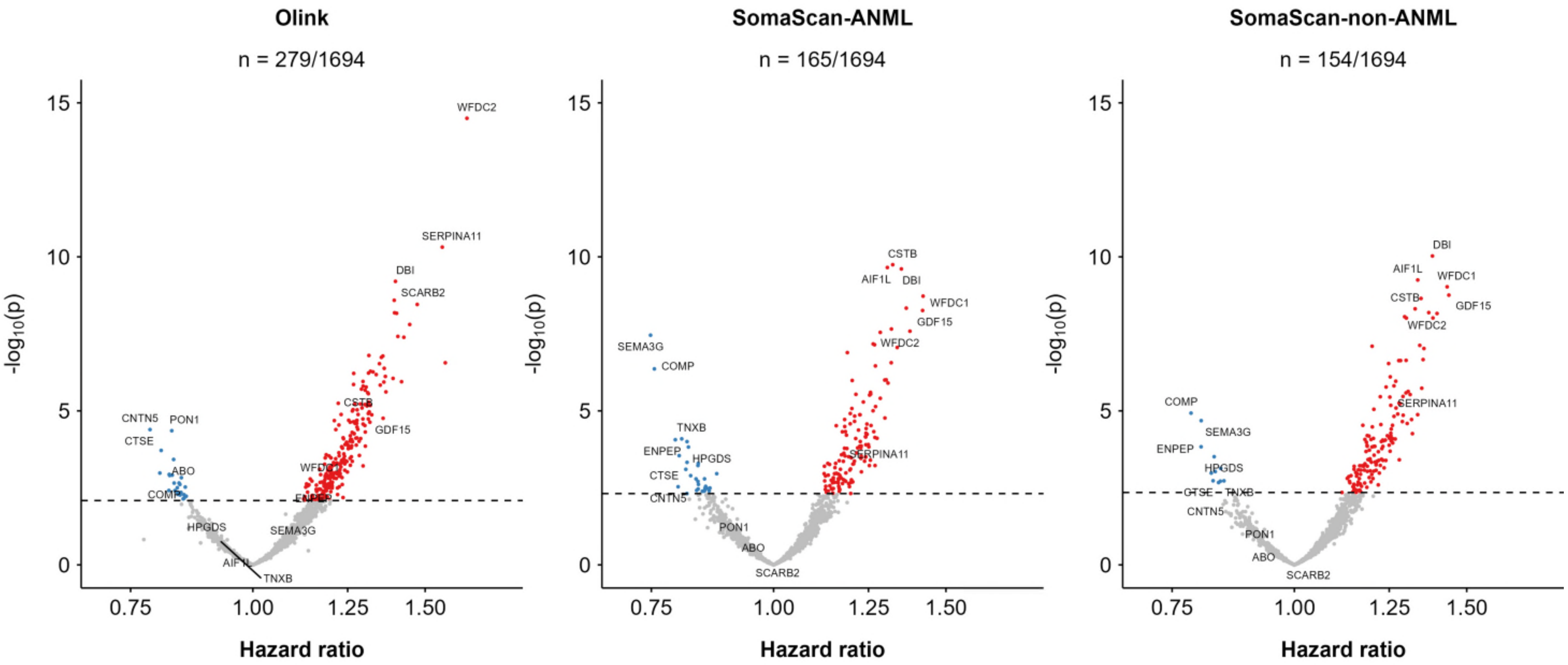

Supplementary Figure 30: Number of proteins significantly associated with incident IHD and their effect sizes after applying Bonferroni correction for multiple testing

Raw p-values shown, with dashed lines indicating Bonferroni corrected significant thresholds.

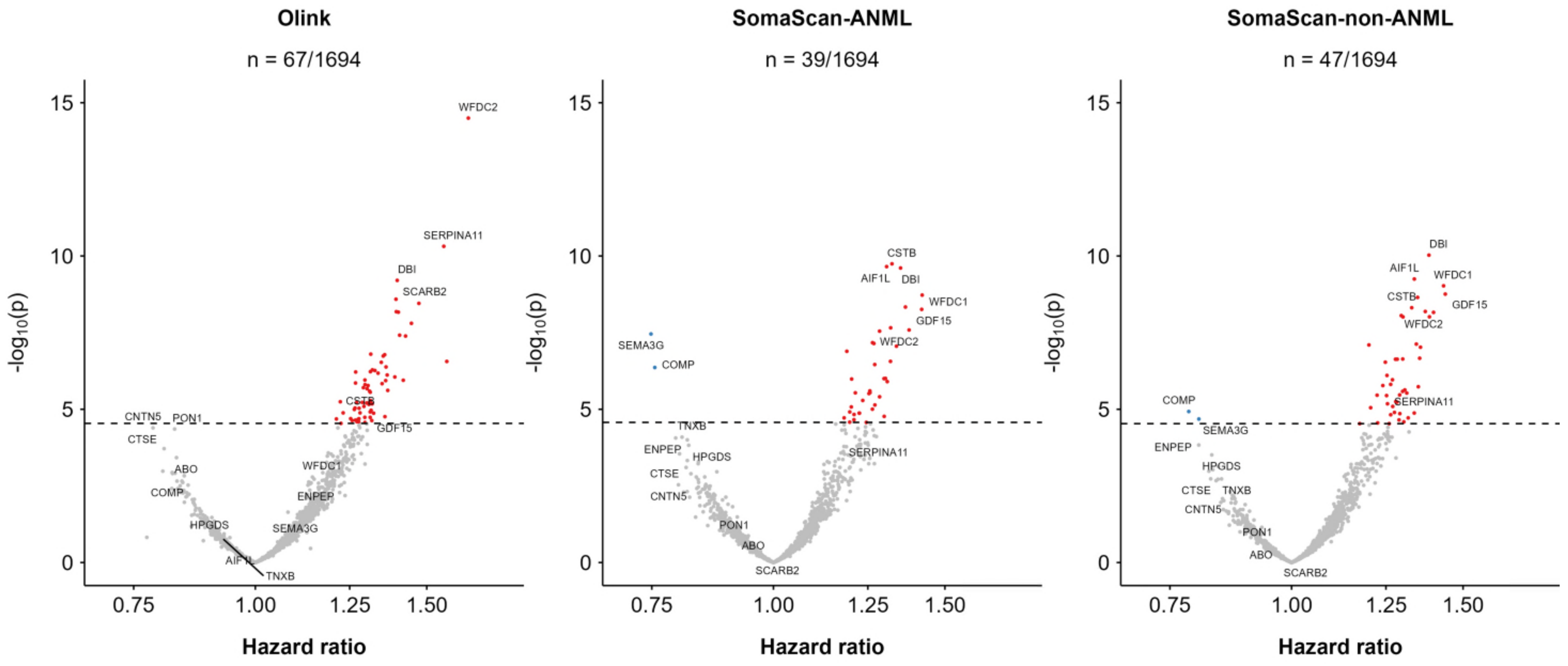

Supplementary Figure 31: Comparison of effect sizes for proteins associated with risk of incident IHD between Olink and SomaScan

Dark dots indicate shared associations between Olink and SomaScan, which were defined as significant associations found in both datasets that were also directionally consistent. Results were corrected using false discovery rate.

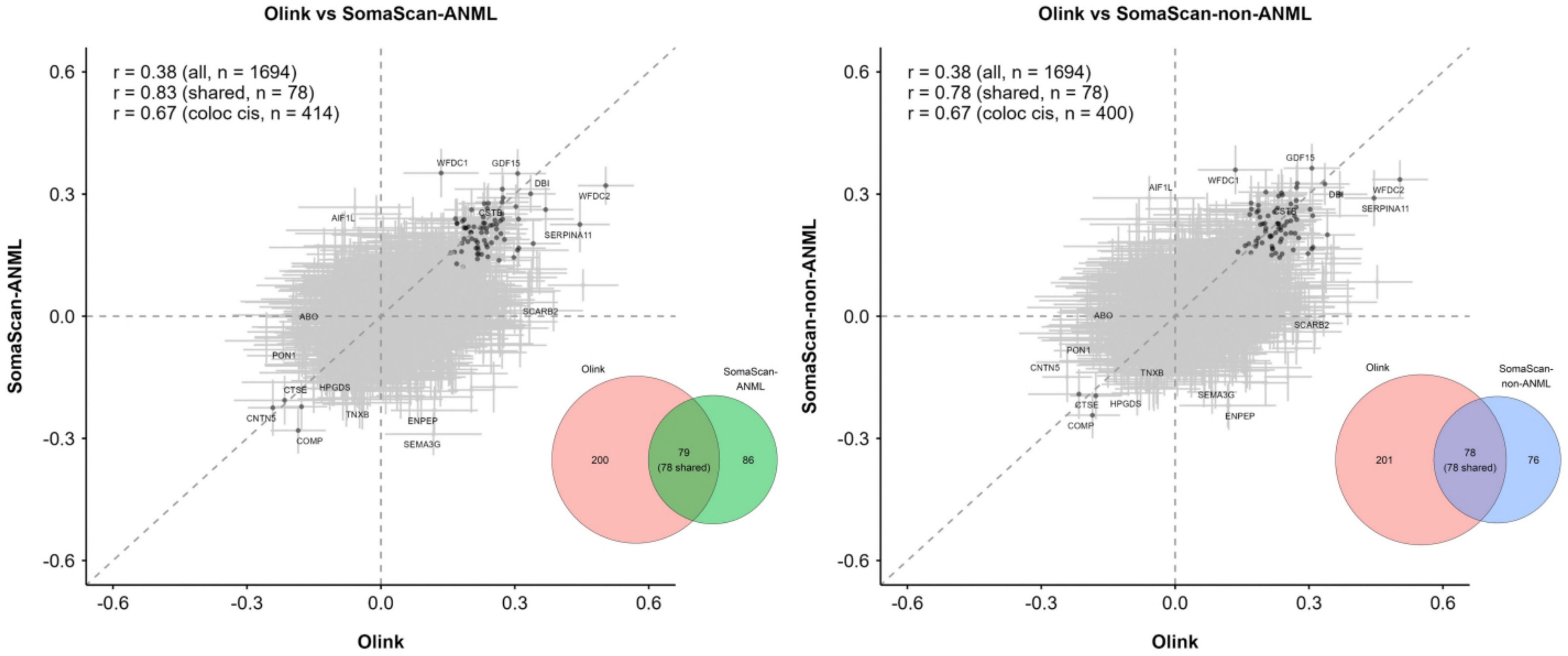

Supplementary Figure 32: Observational correlations and shared associations for IHD

Results were corrected for multiple testing using false discovery rate within each platform.

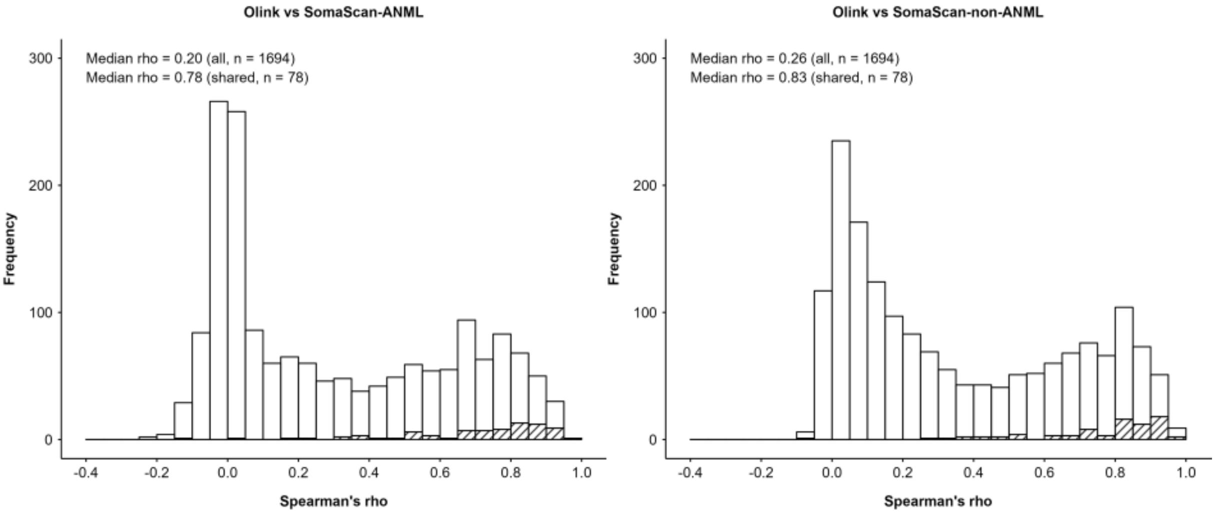

Supplementary Figure 33: Factors explaining discordant findings on IHD associations between Olink and SomaScan

Results were shown based on SomaScan-ANML (A) and SomaScan-non-ANML (B). Proteins were categorised into three groups: proteins associated with IHD in Olink only, proteins associated with IHD in SomaScan only, and proteins associated with IHD in both platforms with the same direction of their effects (shared). There were very few proteins associated with IHD in both platforms and with different directions of their effects. Using shared proteins as the reference group, we tested if observational correlations, key technical factors, or protein-altering variants were could explain discordant findings on IHD associations between platforms. Betas represent effect sizes per SD change for continuous factors, or log odds ratios for binary variables. *: Continuous factors were scaled to show comparable effect sizes in the figures; LOD=limit of detection; QC=quality control; PAV=protein-altering variants.

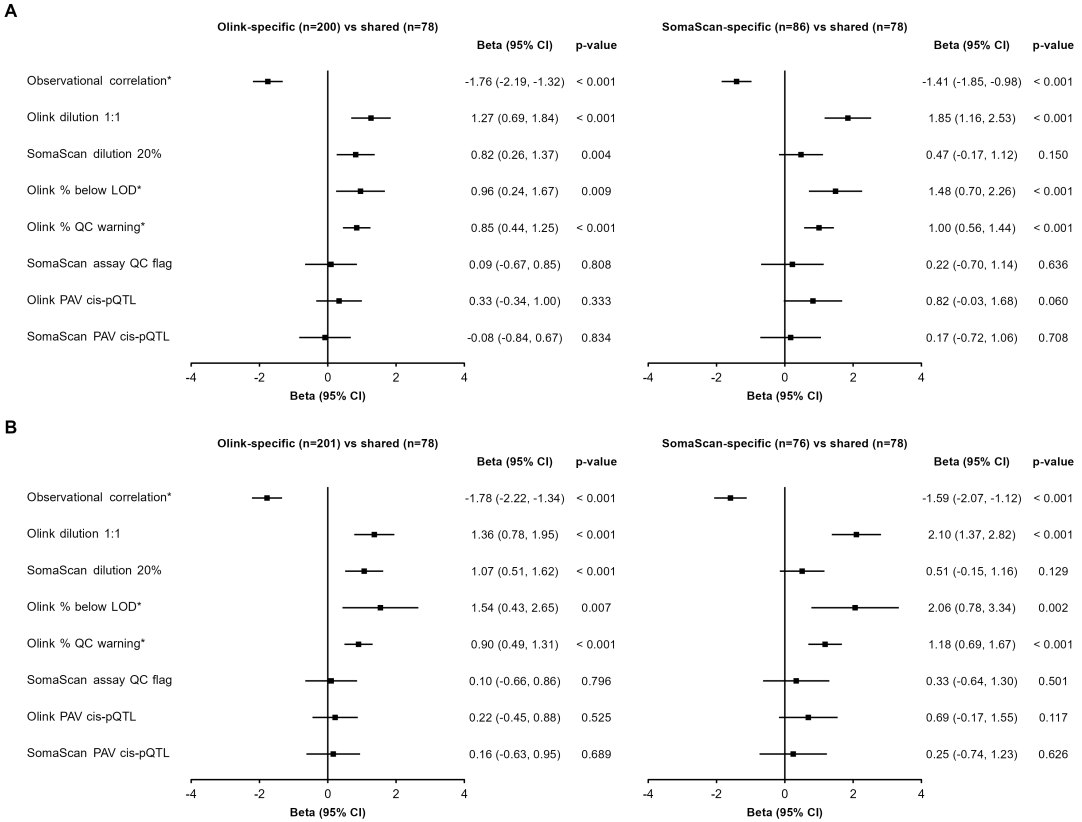

Supplementary Figure 34: Performance of proteins measured using Olink and SomaScan platforms for prediction of incident IHD using the decile-based method

Conventional risk factors for cardiovascular disease included age, sex, smoking, type 2 diabetes, systolic blood pressure, and waist circumference. For each platform, four sets of proteins were used to construct risk prediction models: 1) all overlapping proteins from both platforms; 2) all overlapping proteins from each platform; 3) out of the overlapping proteins, significant proteins after false discovery rate correction; 4) significant proteins that showed directionally consistent effects on IHD risk between Olink and SomaScan. NRI were computed with the decile-based method. Abbreviations: IHD=ischaemic heart disease; NRI=net reclassification index. FDR=false discovery rate.

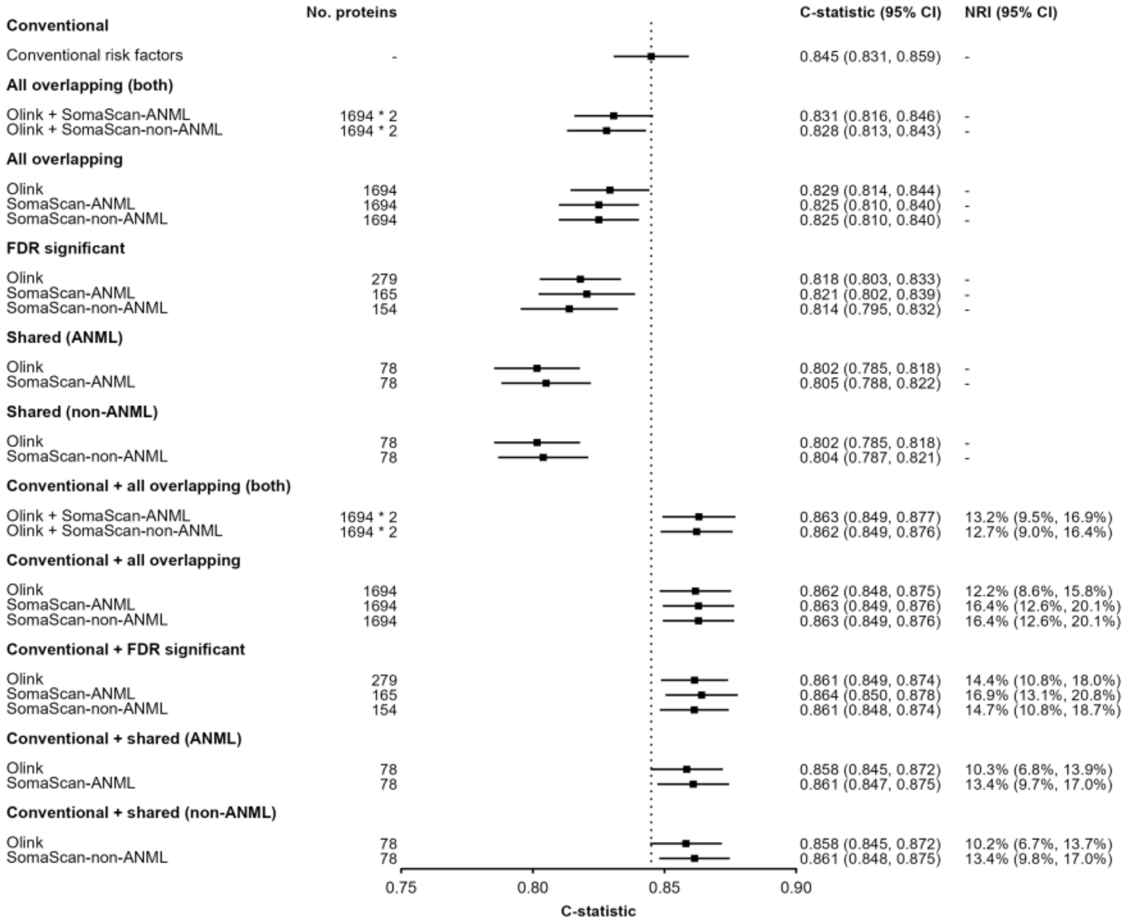

Supplementary Figure 35: Performance of proteins measured using Olink and SomaScan platforms for prediction of incident IHD using the category-free method

Conventional risk factors for cardiovascular disease included age, sex, smoking, type 2 diabetes, systolic blood pressure, and waist circumference. For each platform, three sets of proteins were used to construct risk prediction models: 1) all overlapping proteins from both platforms; 2) all overlapping proteins from each platform; 3) out of the overlapping proteins, significant proteins after false discovery rate correction; 4) significant proteins that showed directionally consistent effects on IHD risk between Olink and SomaScan. NRI were computed with the decile-based method. Abbreviations: IHD=ischaemic heart disease; NRI=net reclassification index. FDR=false discovery rate.

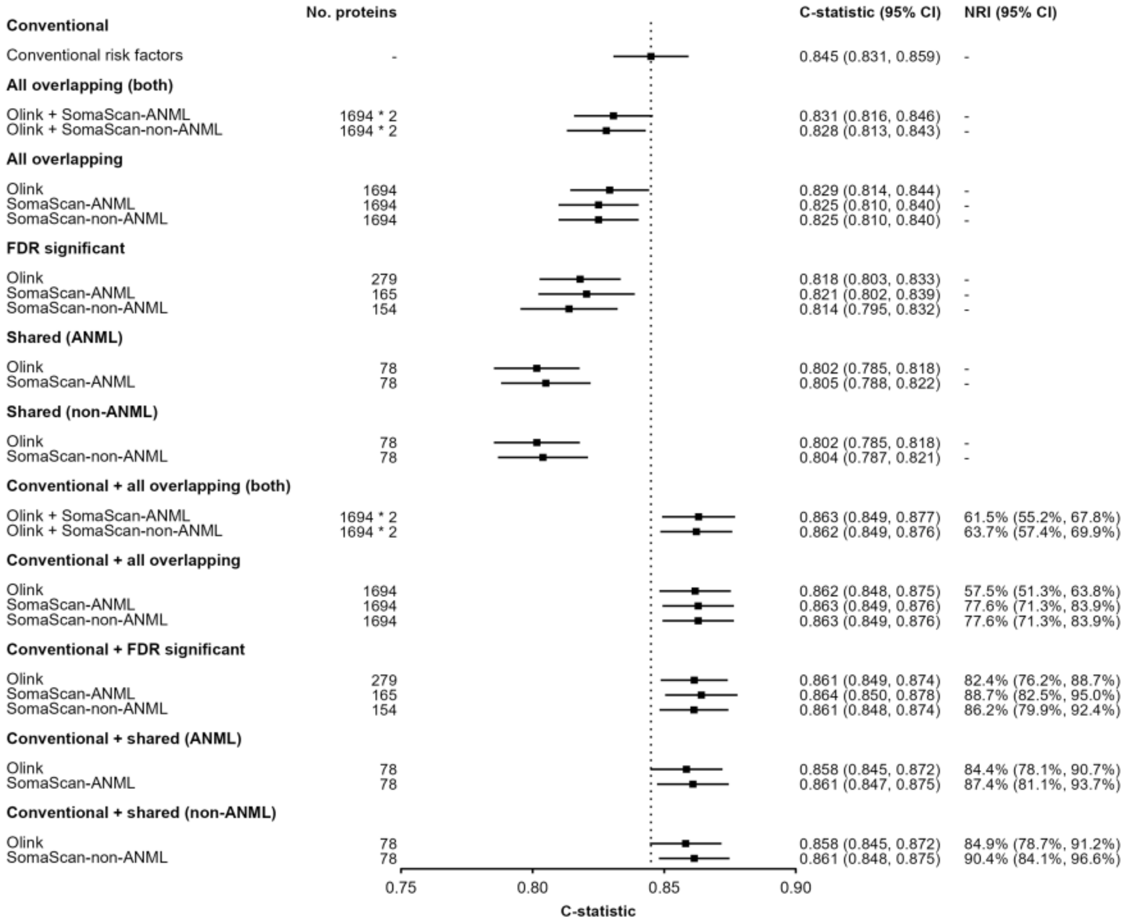

Supplementary Figure 36: Correlations between protein levels measured by SOMAmers targeting the same protein

Analyses conducted on 472 proteins targeted by 2 to 9 SOMAmers (1,037 unique SOMAmers in total).

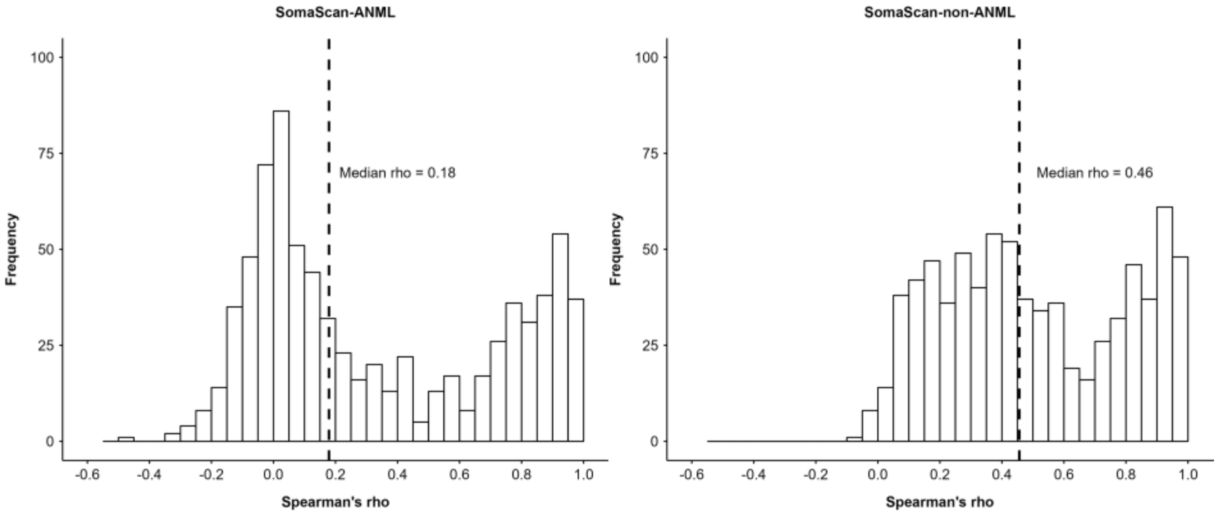

Supplementary Figure 37: Correlations between protein levels measured by Olink-SomaScan reagent pairs involving multiple SOMAmers

Analyses conducted on 472 proteins targeted by one Olink reagent and 2 to 9 SOMAmers (1,037 unique SOMAmers in total), constituting 1,053 Olink-SomaScan reagent pairs.

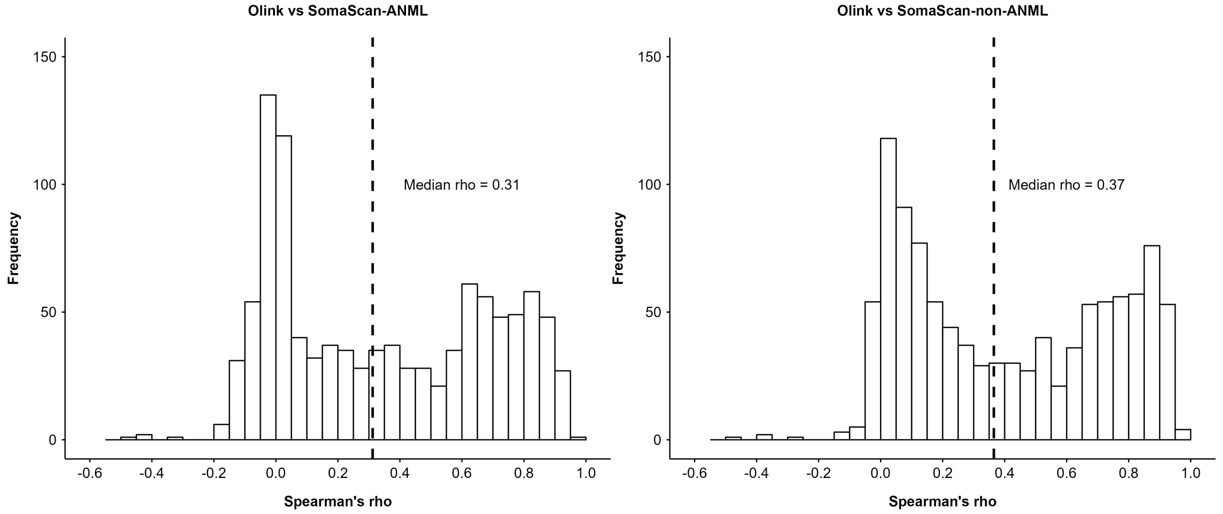

Supplementary Figure 38: Correlations between protein levels measured by Olink reagents targeted by the same SOMAmer

Analyses conducted on 18 pairs of Olink reagents that were matched to the same SOMAmer.

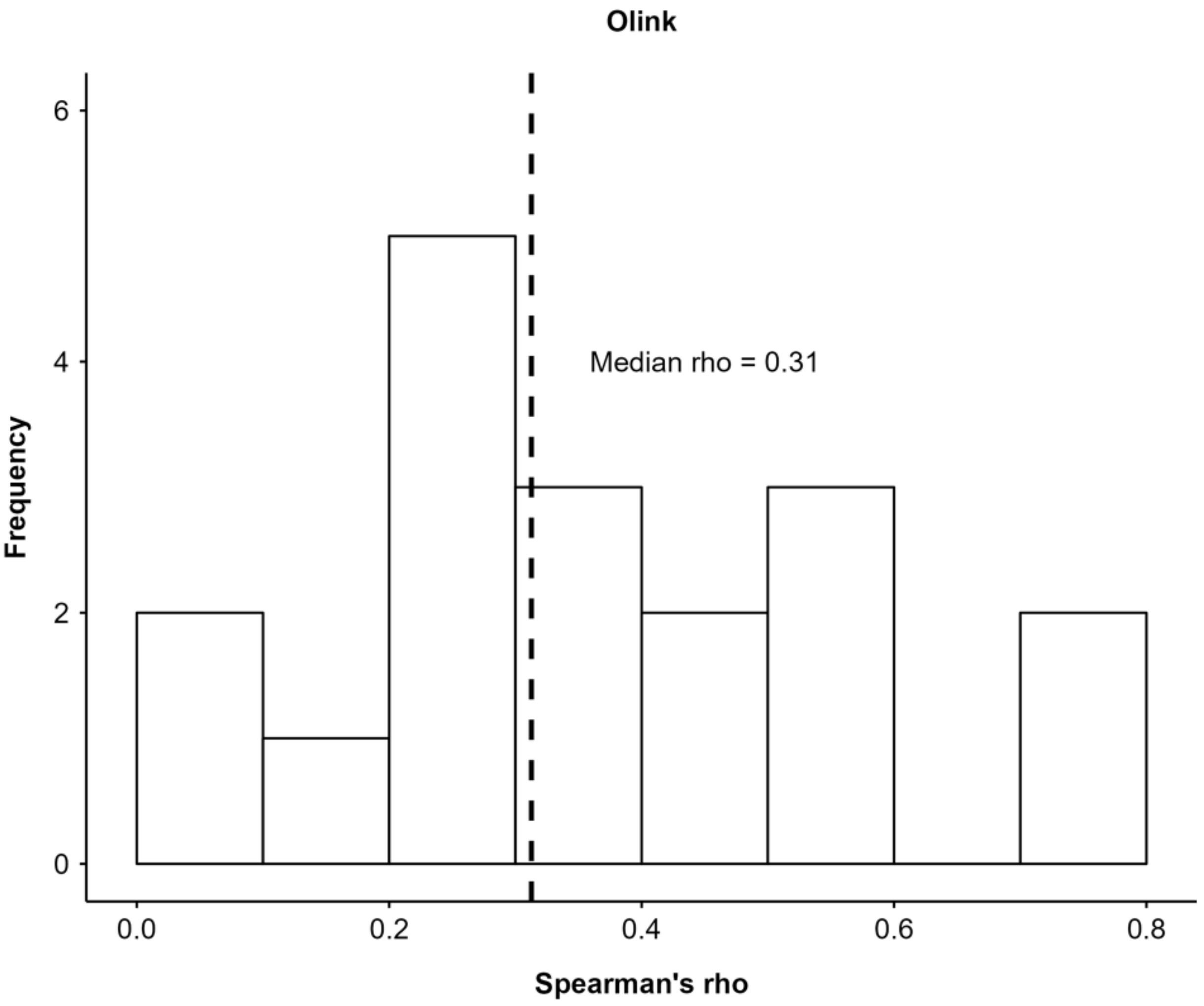

Supplementary Figure 39: Correlations between protein levels measured by Olink-SomaScan reagent pairs involving multiple Olink reagents

Analysis conducted on 36 Olink-SomaScan reagent pairs, involving 18 SOMAmers with each targeting two different proteins.

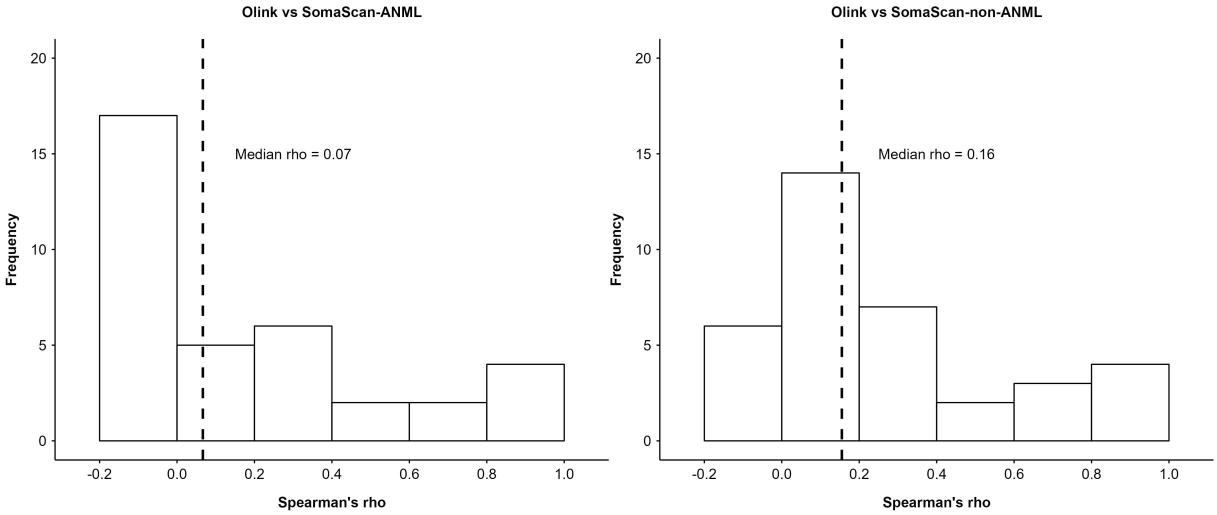

Supplementary Figure 40: Consistency of 1,694 one-to-one matched proteins between Olink and SomaScan based on four criteria

The consistency of the 1,694 one-to-one matched proteins between Olink and SomaScan was assessed based on whether they had i) high observational correlations (rho>0.4), ii) colocalising *cis*-pQTLs, iii) shared associations (significant and directionally consistent associations in both platforms) for BMI, and iv) shared associations for IHD.

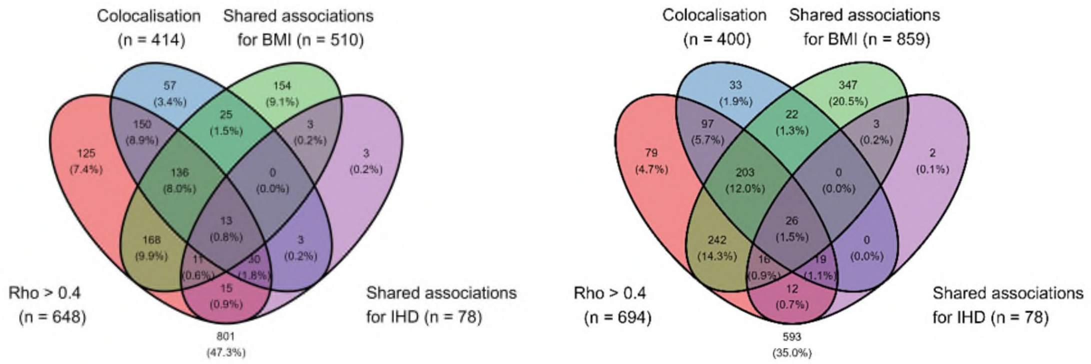

Supplementary Figure 41: An example of colocalisation of *cis*-pQTLs between a SOMAmer targeting TNC and the corresponding Olink reagent

MAF=Minor allele frequency; PP=Posterior probability from colocalisation. Detailed information on *cis*-pQTLs for TNC is shown in Supplementary Data 4.

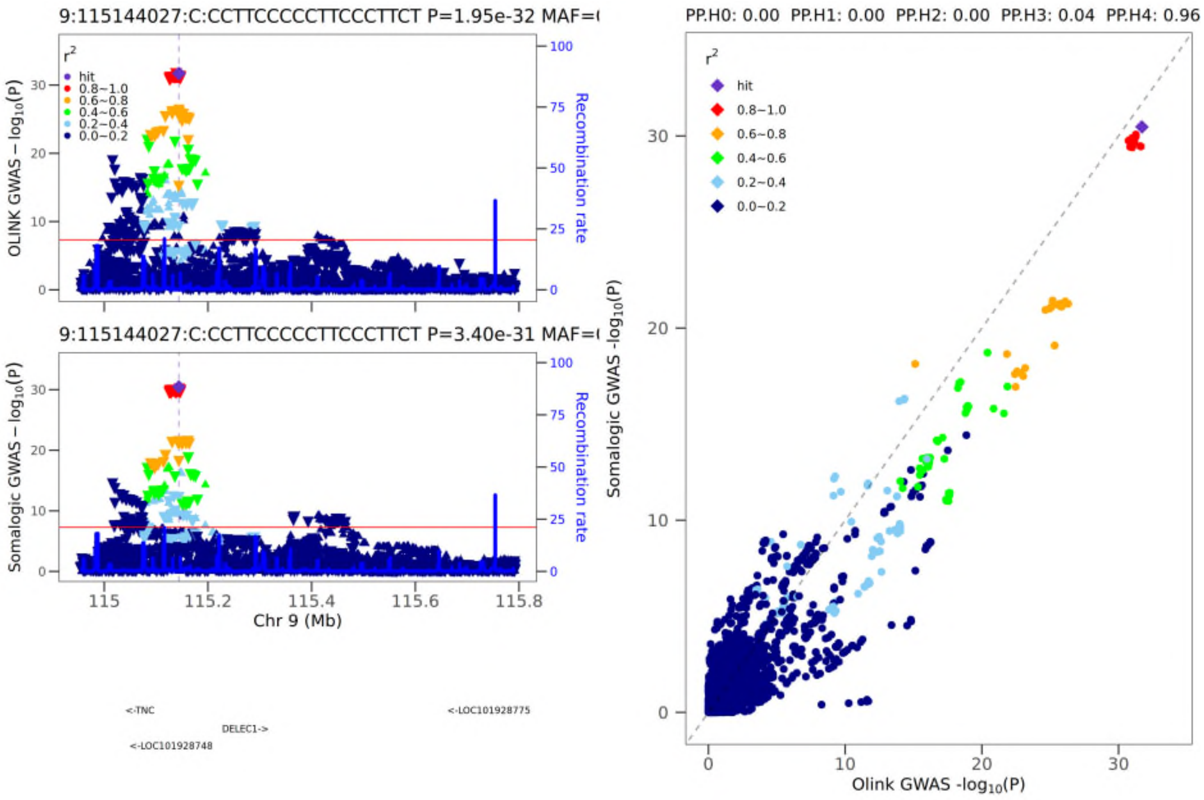

Supplementary Figure 42: An example of no colocalisation of *cis*-pQTLs between a SOMAmer targeting TNC and the corresponding Olink reagent

MAF=Minor allele frequency; PP=Posterior probability from colocalisation. In this example, no *cis*-pQTLs were identified by the SOMAmer. Detailed information on *cis*-pQTLs for TNC is shown in Supplementary Data 4.

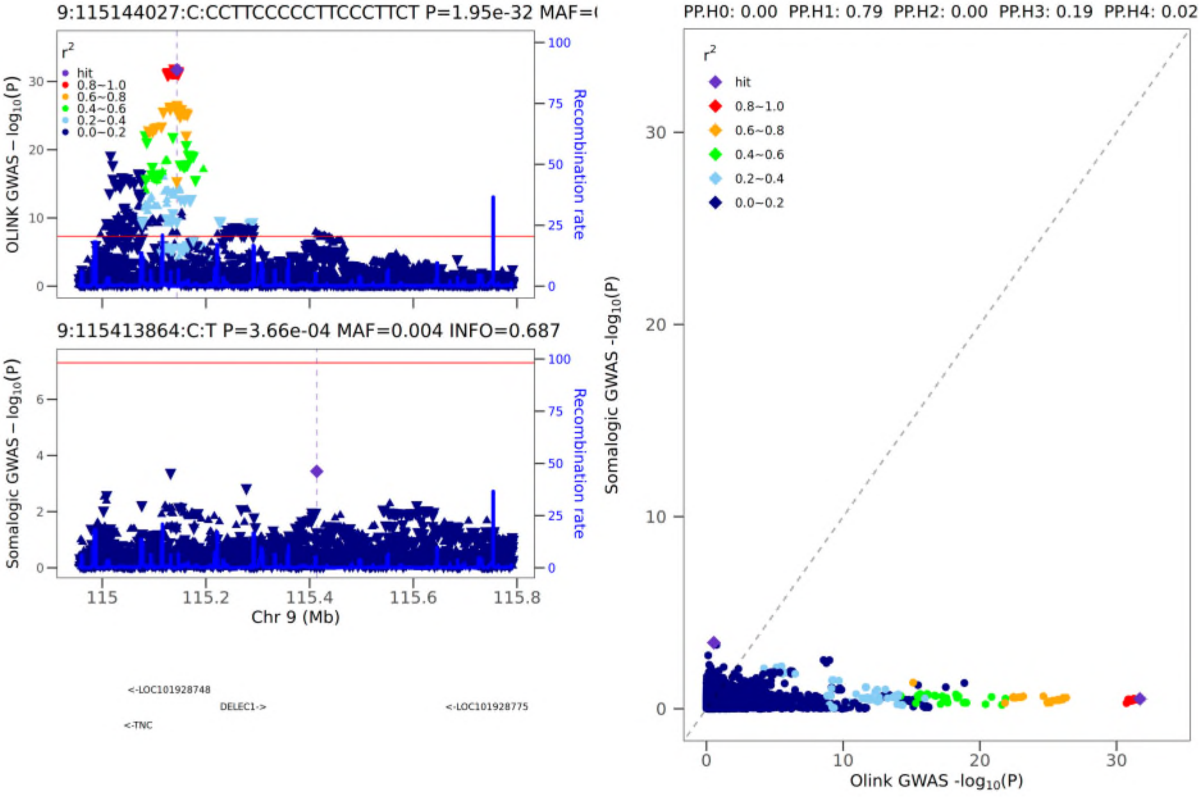

Supplementary Table 1: Protein annotations retrieved from UniProt Knowledgebase for 1,694 overlapping proteins

Protein characteristic	Frequency	Percentage
Initiator methionine	173	10.2
Signal peptide	1,007	59.4
Transit peptide	41	2.4
Propeptide	252	14.9
Peptide	55	3.2
Topological domain	504	29.8
Transmembrane	522	30.8
Repeat	155	9.1
Zinc finger	22	1.3
Coiled coil	99	5.8
Motif	301	17.8
Compositional bias	512	30.2
Active site	357	21.1
Binding site	520	30.7
Non-standard residue	0	0
Modified residue	878	51.8
Lipidation	115	6.8
Glycosylation	936	55.3
Disulfide bond	935	55.2
Cross link	117	6.9
Helix	1,166	68.8
Turn	954	56.3
Beta strand	1,127	66.5
Protein mass (SD), Da	56,736.5	60,773.0
Protein length (SD), residues	512.5	563.3
Number of isoforms (SD)	2.3	1.9

Supplementary Table 2: Top ten most annotated terms under each Gene Ontology (GO) category for 1,694 overlapping proteins

GO term	Frequency	Percentage
**Biological process**		
signal_transduction_go_0007165	214	12.6
cell_adhesion_go_0007155	165	9.7
positive_regulation_of_cell_population_proliferation_go_0008284	148	8.7
proteolysis_go_0006508	138	8.1
inflammatory_response_go_0006954	135	8.0
immune_response_go_0006955	126	7.4
positive_regulation_of_transcription_by_rna_polymerase_ii_go_0045944	106	6.3
negative_regulation_of_apoptotic_process_go_0043066	103	6.1
positive_regulation_of_gene_expression_go_0010628	101	6.0
innate_immune_response_go_0045087	98	5.8
**Cellular component**		
plasma_membrane_go_0005886	692	40.9
extracellular_region_go_0005576	635	37.5
extracellular_space_go_0005615	581	34.3
cytosol_go_0005829	511	30.2
extracellular_exosome_go_0070062	469	27.7
cytoplasm_go_0005737	461	27.2
membrane_go_0016020	328	19.4
nucleus_go_0005634	298	17.6
nucleoplasm_go_0005654	228	13.5
cell_surface_go_0009986	222	13.1
**Molecular function**		
identical_protein_binding_go_0042802	266	15.7
calcium_ion_binding_go_0005509	169	10.0
metal_ion_binding_go_0046872	140	8.3
signaling_receptor_binding_go_0005102	136	8.0
protein_homodimerization_activity_go_0042803	126	7.4
atp_binding_go_0005524	101	6.0
growth_factor_activity_go_0008083	95	5.6
cytokine_activity_go_0005125	87	5.1
integrin_binding_go_0005178	84	5.0
rna_binding_go_0003723	82	4.8

Supplementary Table 3: Number of proteins by their number of matched SOMAmers and colocalisation results (ANML)

No. matched SOMAmers	No. proteins	No. proteins with colocalising *cis*-pQTLs between OLINK and SomaScan					
		Total	Coloc with 1 SOMAmer	Coloc with 2 SOMAmers	Coloc with 3 SOMAmers	Coloc with 4 SOMAmers	Coloc with 6 SOMAmers
2	399	168	84	84	-	-	-
3	56	28	12	9	7	-	-
4	11	9	2	0	3	4	-
5	1	0	0	0	0	0	-
6	2	1	0	0	0	0	1
8	1	1	0	1	0	0	0
9	2	1	0	0	1	0	0

Supplementary Table 4: Number of proteins by their number of matched SOMAmers and colocalisation results (non-ANML)

No. matched SOMAmers	No. proteins	No. proteins with colocalising *cis*-pQTLs between OLINK and SomaScan				
		Total	Coloc with 1 SOMAmer	Coloc with 2 SOMAmers	Coloc with 3 SOMAmers	Coloc with 4 SOMAmers
2	399	165	87	78	-	-
3	56	27	11	9	7	-
4	11	9	1	1	3	4
5	1	0	0	0	0	0
6	2	1	0	0	1	0
8	1	1	0	1	0	0
9	2	1	0	0	0	1
