# Supplementary material for: Comparative studies of 2168 plasma proteins measured by two affinity-based platforms in 4000 Chinese adults
Source: Nat Commun. 2025 Feb 21;16:1869. doi: 10.1038/s41467-025-56935-2 (PMC11845630; doi:10.1038/s41467-025-56935-2)
Supplement: Supplementary file 3 — Description of Additional Supplementary Files [file 41467_2025_56935_MOESM3_ESM.docx]

**Supplementary Data Files**

Supplementary Data 1: This table shows the observational correlations of all matched reagents between OLINK and SomaScan.

Supplementary Data 2: The tables show the pairs of reagents that were mutual best hits (i.e. where they were each other’s most correlated reagent from the other platform).

Supplementary Data 3: This table summarises the number of cis/trans-pQTLs identified in each platform, and the number of colocalising pQTLs across platforms. The r2 columns indicate whether or not the reagent pairs had at least one cis-pQTL pair with r2 > 0.6. The pav columns indicate the number of sentinel cis-pQTLs that were in LD with protein-altering variants.

Supplementary Data 4: This table includes the sentinel (lead) pQTL variants for the overlapping proteins measured by both Olink and SomaScan in CKB. SomaScan pQTLs are based on non-ANML data.

Supplementary Data 5: This table shows the results of proteomic associations with selected baseline traits in CKB. es: effect size. se: standard error.

Supplementary Data 6: This table summarises the results of associations between protein levels and incident IHD. hci: higher confidence interval. lci: lower confidence interval. hr: hazard ratio.

Supplementary Data 7: This table summarises the consistency of protein measurements between Olink and SomaScan, based on whether they had 1) high observational correlations (rho > 0.4), 2) colocalising cis-pQTLs, 3) shared associations for BMI (significant in both platforms with the same direction of effect), and 4) shared associations for IHD (significant in both platforms with the same direction of effect).
